# Orange peel magnetic activated carbon for removal of acid orange 7 dye from water

**DOI:** 10.1038/s41598-023-50273-3

**Published:** 2024-01-02

**Authors:** Asmaa Khalil, Chirangano Mangwandi, Mohamed A. Salem, Safaa Ragab, Ahmed El Nemr

**Affiliations:** 1 Department of Chemistry, Faculty of Science, Tanta University, Tanta, Egypt; 2 School of Chemistry and Chemical Engineering, David Kier Building Queen’s University Belfast, Belfast, BT95AG UK; 3 Environment Divisions, National Institute of Oceanography and Fisheries (NIOF), Kayet Bey, Elanfoushy, Alexandria Egypt

**Keywords:** Pollution remediation, Pollution remediation

## Abstract

Magnetic activated carbon resources with a remarkably high specific surface area have been successfully synthesized using orange peels as the precursor and ZnCl_2_ as the activating agent. The impregnation ratio was set at 0.5, while the pyrolysis temperature spanned from 700 to 900 °C. This comprehensive study delved into the influence of activation temperatures on the resultant pore morphology and specific surface area. Optimal conditions were discerned, leading to a magnetic activated carbon material exhibiting an impressive specific surface area at 700 °C. The Brunauer–Emmett–Teller surface area reached 155.09 m^2^/g, accompanied by a total pore volume of 0.1768 cm^3^/g, and a mean pore diameter of 4.5604 nm. The material displayed noteworthy properties, with saturation magnetization (Ms) reaching 17.28 emu/g, remanence (Mr) at 0.29 emu/g, and coercivity (Hc) of 13.71 G. Additionally, the composite demonstrated super-paramagnetic behaviour at room temperature, facilitating its rapid collection within 5 s through an external magnetic field. Factors such as absorbent dose, initial concentration of the adsorbate, contact time, and pH were systematically examined. The adsorption behaviour for acid orange 7 (AO7) was found to adhere to the Temkin isotherm models (*R*^2^ = 0.997). The Langmuir isotherm model suggested a monolayer adsorption, and the calculated maximum monolayer capacity (*Q*_m_) was 357.14 mg/g, derived from the linear solvation of the Langmuir model using 0.75 g/L as an adsorbent dose and 150–500 mg/L as AO7 dye concentrations. The pseudo-second order model proved to be the best fit for the experimental data of AO7 dye adsorption, with a high coefficient of determination (*R*^2^) ranging from 0.999 to 1.000, outperforming other kinetic models.

## Introduction

Wastewater polluted with dyes from textile and printing industries is a hazard to our health and environment^1^. The textile industry is known for using a lot of water in its manufacturing operations, which results in vast volumes of coloured effluents containing a lot of organic compounds^2^. After dying, around 15% of dye waste is released into the environment. Every year, around 280,000 tons of textile dyes are released into the environment, most of which end up in the ocean and marine environment^3^. Dyes present in water streams affect photosynthesis by reducing the penetration of light causing damage to aquatic life. In some situations, anaerobic breakdown of the dye solution can result in the formation of potentially carcinogenic chemicals that can enter the food chain^4,5^. In the context of biochemical oxygen demand, the prioritization of color removal from wastewaters often outweighs the removal of soluble organic compounds that lack color, as indicated by^4^. Therefore, it must be eliminated before being released into water bodies^6,7^. Because of their beautiful colour, convenience of use, and economic feasibility in terms of synthesis, azo dyes are the most well-liked dyes when compared to other varieties. They are classified as refractory chemical species because they have one or more azo groups (–N=N–), and they are infamous for having toxic, cancer-causing, and allergenic properties^8,9^.

Acid Orange 7 (AO7) is a mono azo dye that is frequently used for dyeing a wide range of materials such as aluminium, nylon, cosmetics, detergents, silk, and wool. This is so because it is water soluble, inexpensive and dyes rather quickly (in a short time) in weak acidic solution. Like the majority of other azo dyes, acid orange 7 dye is disposed of in industrial effluent and is hazardous to human health^10,11^. Highly perilous, ingestion of this substance can lead to skin, eye, and mucous membrane irritation, along with affecting the upper respiratory system. It may also result in intense feelings of nausea, headaches, and dizziness. Additionally, it results in bone marrow loss, which can induce anaemia. Consuming it might also be fatal due to its cancer-causing and tumour-causing properties^12,13^. When the azo group is deficient in electrons, the AO7 dye has a tendency to pull those electrons away, turning the azo group into the carcinogenic amino compounds [1-amino-2-naphthol] that have been associated with bladder tumours^10,11^. The majority of countries enforce stringent regulations concerning the disposal of wastewater containing AO7 azo dyes. This is driven by concerns related to both aesthetic aspects and the presence of dangerous and mutagenic by-products that can result from their breakdown^12^.

To remove various pollutants from textile industry wastewater, various removal techniques such as chemical oxidation and reduction^13^, membrane filtration^14^, photo-catalysis^15^, ozonation^16^, coagulation electrochemical treatment^17^, ion exchange^18^, sedimentation^19^, flocculation^20^ and adsorption^21^ techniques can be used. Adsorption, a process involving the transfer of dye molecules onto an adsorbent phase while allowing the clean effluent to remain unaffected, is an economical approach used for dye removal. This method utilizes cost-effective adsorbents and is considered an effective technique. The adsorption method was applied by several research teams to clean up dye-contaminated water^22^, used a chitosan (CS)—cerium oxide (CeO_2_) nano biosorbent to remove indigo carmine from aqueous solutions. Naser et al.^23^ looked at the usage of inexpensive adsorbent material like onion peels to remove the colour methyl violet using a batch adsorption procedure. In another related study used papaya seed were as adsorbent for the removal of methylene blue^24^. In addition, Salem et al.^25^ used polyaniline/Fe_3_O_4_ (PANI/Fe_3_O_4_) nanocomposite as an adsorbent for removal of titan yellow (TY) dye from wastewater. There are other studies reported in literature were low-cost biomass derived adsorbent materials were applied as adsorbent materials for the removal of different range of dyes^26–35^.

For the organic pollutants adsorption, conventional adsorbents such as inorganic clay^36^, organic clay^37^, activated carbon^11,38,39^, MIEX resin^40^, Mg_3_Al LDH^41^ and silica-based^42^, were employed. Activated carbon is the most widely used adsorbent because it has a high adsorption capacity for a range of organic compounds, including heavy metals, dyes, and surfactants^45–52^. The surface chemistry of activated carbons was examined by researchers to interpret dye adsorption^43,44^. Activated carbons were created during the last ten years from a variety of agricultural wastes, including rice husk, sawdust, maize cobs and orange peels. These carbons were used as inexpensive adsorbents to remove various pollutants from aqueous solutions^45^. In the present research, we've opted to utilize orange fruit peel as the primary source for producing extensively porous activated carbon^45,46^. Orange peels are commonly available and economical resources often discarded by fresh juice vendors and fruit stands^47^. According to the FAO in 2012, annual global orange production is predicted to be more than 68 million tonnes, with Egypt accounting for more than 2.7 million tonnes of that total. Orange peel contains 13.61% cellulose, 6.1% hemicellulose, 2.1% lignin, and ash (1.5%)^47^. The magnetic property of activated carbon is introduced through the incorporation of magnetic nanoparticles, such as magnetite (Fe_3_O_4_). This property allows for easy separation of the adsorbent from the solution after adsorption. Magnetic activated carbon can be manipulated using an external magnetic field, making the process more efficient and environmentally friendly^48–51^.

This study aims to make orange peel-activated carbon (activated with ZnCl_2_) then combined with magnetic iron oxide nanoparticles to obtain composite material, magnetic OPAC (MG-OPAC). The incorporated magnetic properties will make it easier to isolate the adsorbent from water using external magnetic field (magnetic separators) and used it as adsorbent for removal of AO7 dye [p-(2-hydroxy-1-napththylazo) benzene sulfonic acid] in a batch system. Utilising a variety of analytical approaches, it was determined how process variables like contact duration, pH, adsorbate concentration, and quantity of adsorbent affected the dye removal efficiency. Studies on the kinetic, isothermal, and thermodynamic aspects of AO7 dye adsorption by MG-OPAC were conducted.

## Materials and methods

### Materials and chemicals

The orange peels for this investigation were sourced from a neighbourhood market in Alexandria, Egypt, washed with distilled water which was followed by drying in an oven for 24 h at 50 °C. The dried peels were blended into a powder, then put away until needed. By combining 1.0 g of AO7 dye with 1 L of double-distilled water, AO7 dye stock solution was created. Ferric nitrate anhydrous (Fe(NO_3_)_3_, M.W 241.86 g, assay 98%) for preparation of magnetite was acquired from ADWIC, El-Nasr Pharm. Chem. Co., Egypt. Ferrous sulphate (FeSO_4.7_H_2_O, M.W 278.01 g, assay 98.5%) for preparation of magnetite Fe_3_O_4_ was obtained from Alpha Chemika, India. Zinc chloride (ZnCl_2_, M.W 136.30 g, assay 99.5%) as chemical activator was procured from Universal Fine Chemicals Pvt-Ltd, Mumbai, India, and hydrochloric acid (HCl, M.W 36.46 g, assay 30–34%) was procured from SD Fine-Chem Limited (SD FCL).

### Synthesis of orange peel activated carbon (OPAC)

Distilled water was used to clean the orange peels and then it was dried for 24 h at 50 °C. The dried orange peels were put through a blender and then activated with ZnCl_2_ at 105 °C for 24 h in a 1:2 (W/W) peels to ZnCl_2_ ratio. Following that, it was heated to 700–900 °C for one hour with nitrogen flowing at a rate of 50 mL/min in a tube furnace (Nabertherm B180 (RT 50/250/13)). It produces carbon in powder form. In order to remove the alkali and minerals and achieve a neutral pH, the activated carbon was cooled to room temperature, refluxed with 1N HCl for two hours, and then washed with distilled water. It was finally dried for 4 h at 110 °C.

### Preparation of magnetic orange peels activated carbon (MG-OPAC)

By suspending (1.0 g) AC in (500 mL) of a solution comprising 3.5 g (8.66 mmoL) Fe(NO_3_)_3_ and 1.3 g (4.33 mmoL) FeSO_4_.7H_2_0, magnetic activated carbon prepared at 700 °C (MG-OPAC) was created. The procedure took place at a temperature of 50 °C, involving intense agitation for 60 min subsequent to a 10-min sonication of the solution (40 W, 200 kHz) to induce iron oxide precipitation. Following this, a NaOH aqueous solution was gradually introduced into the mixture until the pH reached the range of 11 to 12. The resulting precipitate was intermittently washed using double-distilled water until a neutral pH was achieved. The subsequent separation of the precipitate from the aqueous mixture was accomplished either by utilizing an external magnetic field or through filtration. Upon completion of the drying process, the MG-OPAC composite was placed within a sealed container for storage until it was ready for use.

### Artificial wastewater preparation

The stock solution of (1.0 g/L) AO7 dye was obtained by dissolving 1.0 g of AO7 dye in deionized water and diluting to 1 L. The working solutions of AO7 dye were obtained by appropriately diluting this stock solution. Using 0.01M NaOH or HCl, the pH of the liquids was changed. Spectrophotometric analysis was used to calculate the dye concentration at λ_max_ 483 nm. Additionally, from the stock solution, concentrations in the 10 to 150 mg/L were generated in order to create a calibration curve.

### Sample characterization

Utilizing a surface area and pore analyzer (BELSORP—Mini II, BEL Japan, Inc.), the specific surface area was determined by analyzing N_2_ adsorption/desorption isotherms at a temperature of 77.4 K, encompassing a relative pressure (P/P°) span from 0.001 to 1.0. Employing the Brunauer–Emmett–Teller (BET) technique^52,53^, the specific surface area (SBET), total pore volume (V_T_), and average pore diameter (D_P_) of the produced composites were determined. Moreover, micropore surface area (S_mi_) and micropore volume (V_mi_) were calculated using the t-plot approach and BELSORP analysis program software. For characterization of functional groups within activated carbon (AC), magnetic activated carbon prepared at 700 °C (MG-OPAC), and the MG-OPAC composite post-AO7 dye removal, Fourier transform infrared (FT-IR) spectroscopy was conducted using a Bruker VERTEX70 equipped with a platinum ATR type V-100. The wave number range of 400–4000 cm^–1^ was examined.

The composite's morphology was investigated through scanning electron microscopy (SEM), and the components present on the sample surfaces were identified using energy-dispersive X-ray analysis (EDX). SEM and EDX images were captured using the Quanta FEG 250, a UK-manufactured instrument. To assess the crystalline properties of MG-OPAC, an X-Ray diffractometer (Panalytica) employing Cu K radiation (k = 0.15406 nm) and scanning within the range of 2Ɵ (0—90) was employed.

To evaluate the magnetic characteristics of the composite materials at room temperature, Vibrating Sample Magnetometers (VSM) from VSM Lake Shore model 7410 in the USA were employed.

### Batch adsorption study

The effect of several variables such as the amount of adsorbent, pH levels, and the duration of contact between the adsorbent and adsorbate, were investigated using batch experiment. MG-OPAC was used as the adsorbent in 100 mL of AO7 dye solution batch adsorption studies, which were conducted at (25 °C) and 200 rpm. When the appropriate contact time had passed, the flask was removed, centrifuged, and the remaining concentration of the AO7 dye was calculated using a UV/VIS spectrophotometer (Labomed UVD 3500) at λ_max_ = 483 (Concentration = 21.854 × Adsorption). On the removal of AO7 dye, the effects of adsorbent dose (0.75–3.5 g/L), contact period (15–180 min), and starting adsorbate (AO7 dye) concentration (150–500 mg/L) were examined. The following Eqs. (1), (2) were used to compute the equilibrium adsorption capacity of the adsorbent, *q*_e_ (mg/g).

1 $${q}_{e}=\frac{\left({C}_{i}-{C}_{e}\right)}{m}V$$

2 $$R\%=\frac{100\left({C}_{i}-{C}_{e}\right)}{{C}_{i}}$$

where ${C}_{i}$ is primary adsorbate concentration, ${C}_{e}$ is the residual concentration reached at equilibrium state, $V$ is the volume of solution (L), $R\%$ is the removal percent and $m$ is the mass of adsorbent (g).

## Results and discussion

### BET surface area

**Figure 1:**
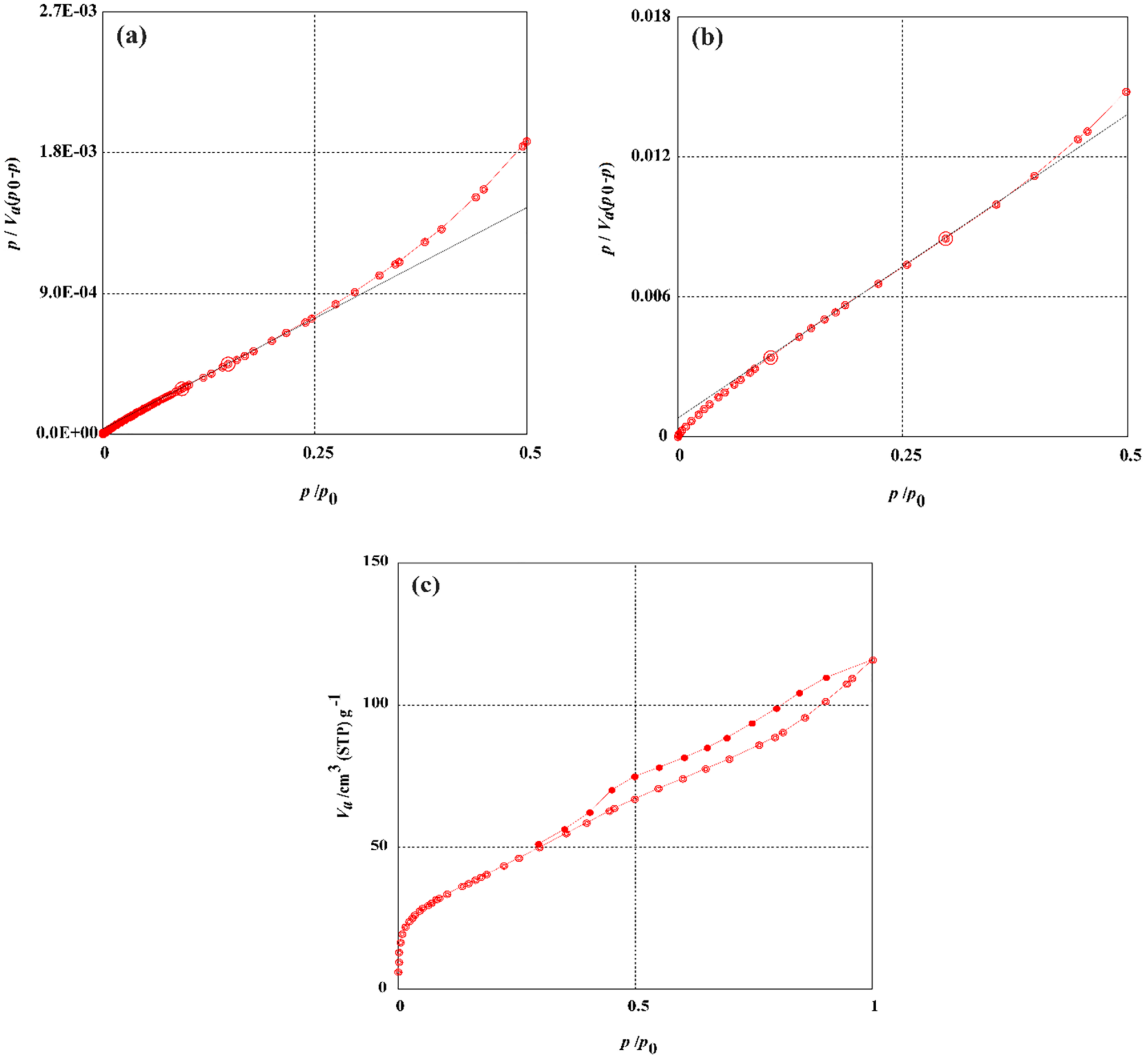
(**a**) BET analysis curve of OPAC prepared at 700 °C, (**b**) BET analysis curve of MG-OPAC prepared at 700 °C, (**c**) Adsorption ()-desorption () analysis curve of MG-OPAC prepared at 700 °C.

The carbonization temperature impact on preparation of OPAC was investigated at pyrolysis temperatures ranged between 700 and 900 °C. As depicted in Table 1, the specific surface area and pore volume exhibit a noteworthy trend in response to varying carbonization temperatures. At a pyrolysis temperature of 700 °C (Fig. 1a), the OPAC sample displayed the highest specific surface area of 1512.5 m^2^/g and pore volume of 0.8854 cm^3^/g. This can be attributed to the ideal conditions for carbonization at this temperature, facilitating the optimal development of micropores and mesopores within the activated carbon structure. However, as the carbonization temperature is elevated to 900 °C, a gradual decline in both surface area and pore volume is observed. The reduction in these parameters can be linked to the heat-induced shrinkage of pores, leading to a decrease in the available pore surface area^54^. This trend was also confirmed by Wu et al. (2022), in the production of magnetic biochars from teawaste^48^.

**Table 1:** Effect of temperature on Surface area and pore volumes of OPAC prepared at 700, 800, and 900 °C.

Method sample	Temperature (°C)	BET			*t*-plot	
		*S*_BET_ (m^2^ g)	*V*_T_ (cm^3^/g)	*D*_P_ (nm)	*S*_mi_ (m^2^/g)	*V*_mi_ (cm^3^/g)
OPAC	700	1512.5	0.8854	2.3415	1474.0	0.8624
	800	1472.1	0.8411	2.2855	1444.1	0.8147
	900	1411.6	0.9506	2.6935	1323.9	0.9211

The inclusion of iron oxide (Fe_3_O_4_) across the porous surface of OPAC resulted in lower surface areas (*S*_BET_) 155.09 m^2^/g, total pore volume (*V*_T_) 0.1768 cm^3^/g, and mean pore diameter (*D*_P_) 4.5604 nm (Fig. 1b) for the generated magnetic composite (MG-OPAC) prepared from magnetic activated carbon formed at 700 °C. The N_2_ adsorption–desorption isotherms of produced MG-OPAC are described in Fig. 1c. In accordance with the IUPAC categorization, the sample displayed step type IV isotherms^55,56^. The knee formation implies monolayer-coated micropores, the observed plateau shows multilayer adsorption through the pores at moderate pressures, and the isotherm IV reveals that adsorbate gas fills micropores at relatively low pressures. On the other hand, the capillary condensation that occurred in the mesopores was made clear by the increase in adsorbed volume at high relative pressure.

### XRD

**Figure 2:**
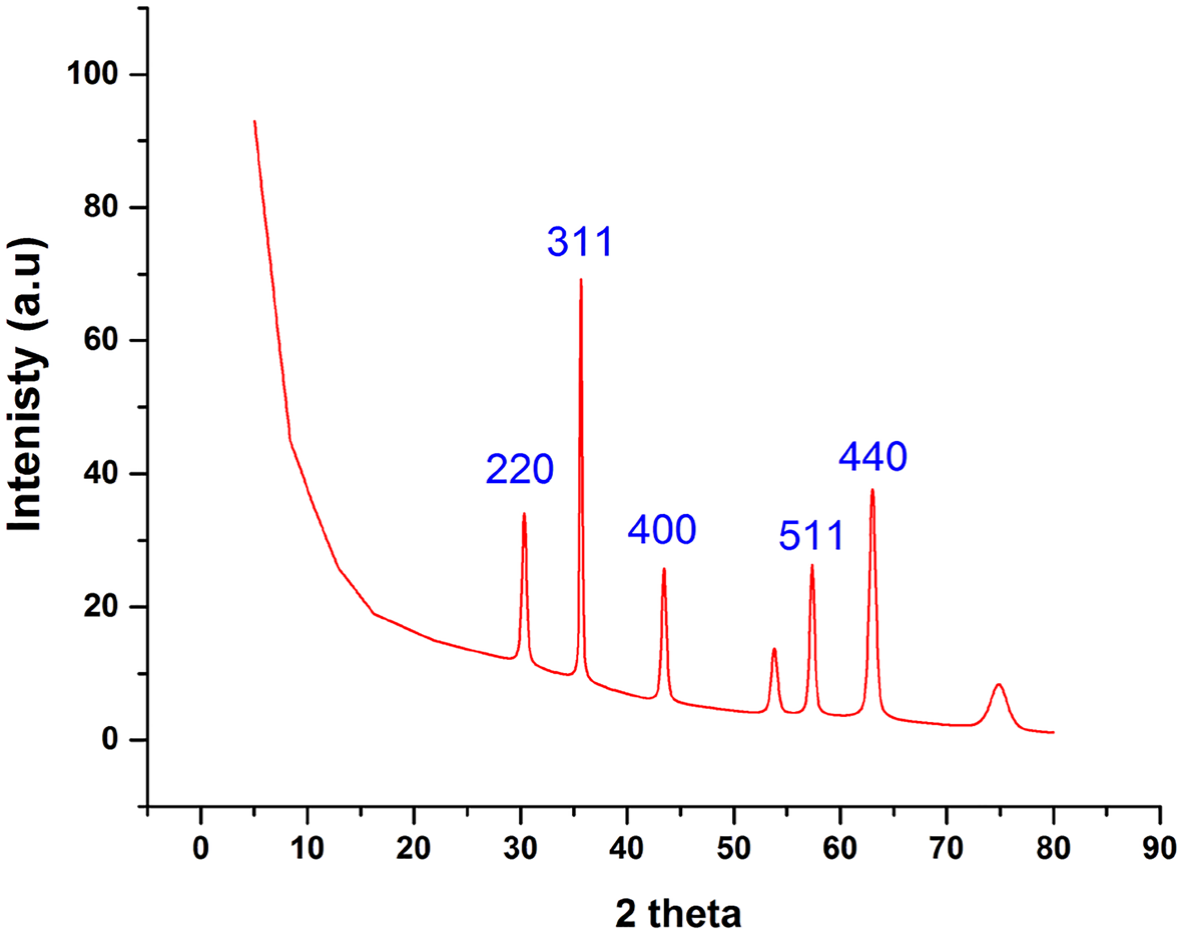
XRD pattern of the formed magnetic-orange peels activated carbon (MG-OPAC).

Powder X-ray diffraction analysis was used to evaluate the samples in order to confirm that the iron oxide in the porous carbon sample (Fe_3_O_4_) to be magnetised is in the crystalline phase. The XRD diffractogram pattern of iron oxide isolated from a solution of ferric chloride and ferrous sulphate (FeSO_4_) is shown in Fig. 2. According to card NO 00-001-1111, the synthesised iron oxide-activated carbon's obtained XRD pattern is shown in Fig. 2. The pattern shows three intense peaks assigned to Fe_3_O_4_ has 220, 311, 400, 511, and 440 faces, which are represented by the observed peaks at 2*Ɵ* = 30.1°, 35.5°, 43°, 57.2°, and 62.7°, respectively. Fe_3_O_4_ and activated carbon composite production was validated by the acquired XRD pattern^57^. For comparison, the XRD analysis of OPAC prepared at 700 °C under N2 was presented in Fig. S1 in the supplement data.

### IR analysis

**Figure 3:**
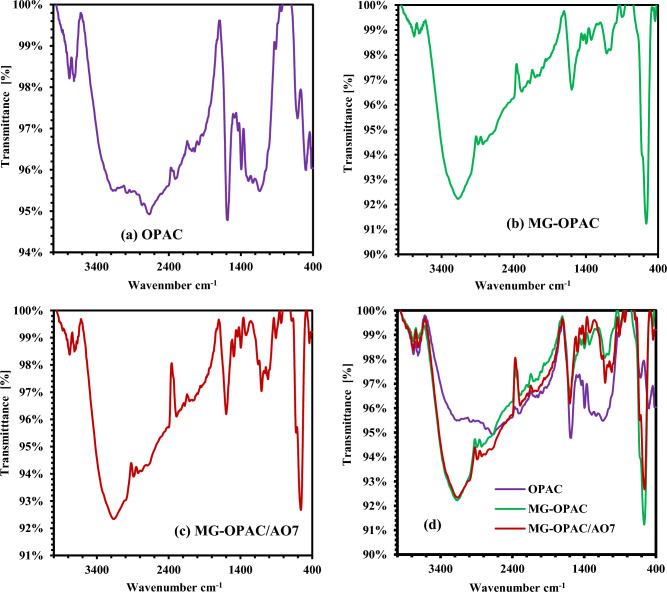
FTIR spectrum of (**a**) OPAC prepared at 700 °C, (**b**) MG-OPAC prepared at 700 °C, (**c**) AO7 dye adsorbed on MG-OPAC prepared at 700 °C, (**d**) a, b, and c in one image.

The functional groups within activated carbon (OPAC at 700 °C), magnetic activated carbon (MG-OPAC at 700 °C), and the MG-OPAC composite were characterized through Fourier transform infrared (FT-IR) spectroscopy. This analysis was conducted using a Bruker VERTEX70 instrument equipped with a platinum attenuated total reflectance (ATR) model V-100, covering the wave number range of 400–4000 cm^–1^. The detailed peaks of the FTIR was presented in the Fig. S2 in the supplement data. Figure 3a shows the FTIR spectrum for OPAC (at 700 °C): the large peak that can be attributed to O–H boning at (3775.4–3715.9) cm^–1^^58^. Due to the presence of water molecules during the assay, likely brought on by semi-dry materials, the O–H group is present. The peak at 2322.6 and 1583.64 cm^–1^, respectively, was connected to the C-H in the methyl group and the C=C aromatic ring^59^. The sample contains oxygen, which is indicated by the peak at 1393.4 cm^–1^ caused by O–H bending. The C-O stretching of alcohol, phenol, ether, and ester caused the peak at 1137.8 cm^–1^^60^. C–C stretching causes the peak at 644.87 cm^–1^. Figure 3b's spectrum shows the occurrence of a new, strong peak at 563.9 cm^–1^ that may belong to the M–O band and may represent the interaction of iron and oxygen in the samples^49^. The C-O stretching and M–O bands were displaced to (1601.14, 1059.55, and 559.71 cm^–1^) in Fig. 3c peak, which corresponds to the C = C aromatic ring, and these alterations in FTIR spectra corroborate the binding of AO7 dye with functional groups contained in the adsorbent (MG-OPAC at 700 °C).

### VSM analysis

**Figure 4:**
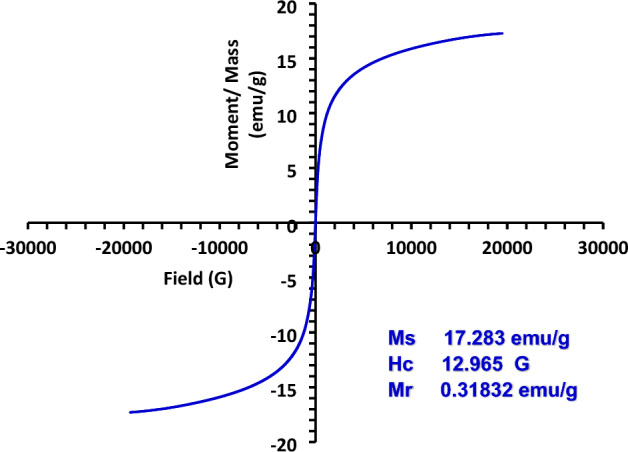
MG-OPAC magnetic hysteresis loop analysis_._

As seen in Fig. 4, the magnetic hysteresis loop of MG-OPAC (OPAC/Fe_3_O_4_) showed almost zero coercivity and remanence, demonstrating the composite's typical paramagnetic performance.

The values of the coercive force (Hc), saturation magnetization (Ms), and remenance (Mr) were determined to be 17.283 emu/g, 0.31832 emu/g, and 12.965 G, respectively. As shown by the low remanence value of less than 25% and the ratio of remanence to saturation magnetization (Mr/Ms = 0.02), the composite was super-paramagnetic at ambient temperature. According to the inset Fig. 4 and an external magnetic field, the composite may be collected in less than 5 S (< 5 S)^50,51^.

### SEM and EDX analysis

**Figure 5:**
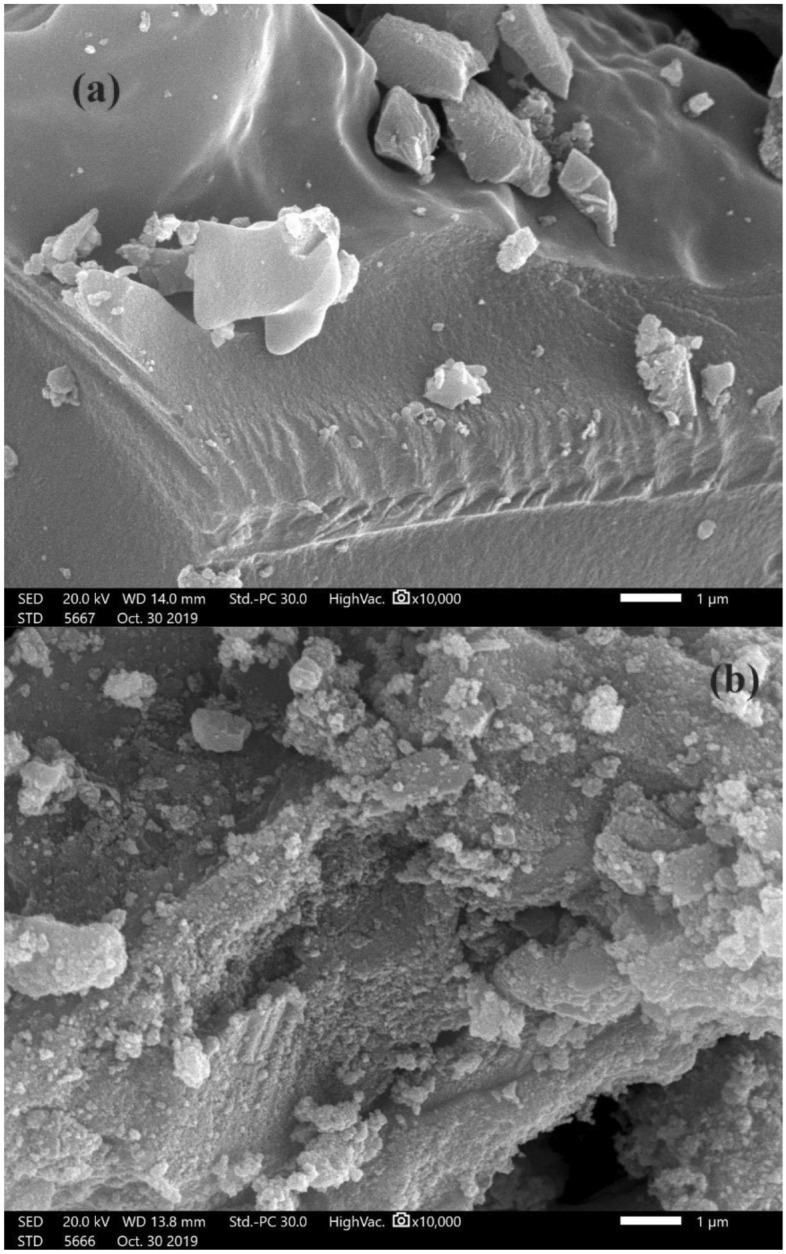
High vacuum SEM analysis image of (**a**) OPAC, (**b**) MG-OPAC under magnification 10,000.

In order to show the surface morphology of the materials used in the creation of activated carbon and magnetic activated carbon, scanning electron microscopy (SEM) examination was employed. Fe_3_O_4_ was deposited onto the surface of OPAC to create MG-OPAC, as shown in Fig. 5b. The TEM image of MG-OPAC was presented in Fig. S3 in the supplement data.

**Figure 6:**
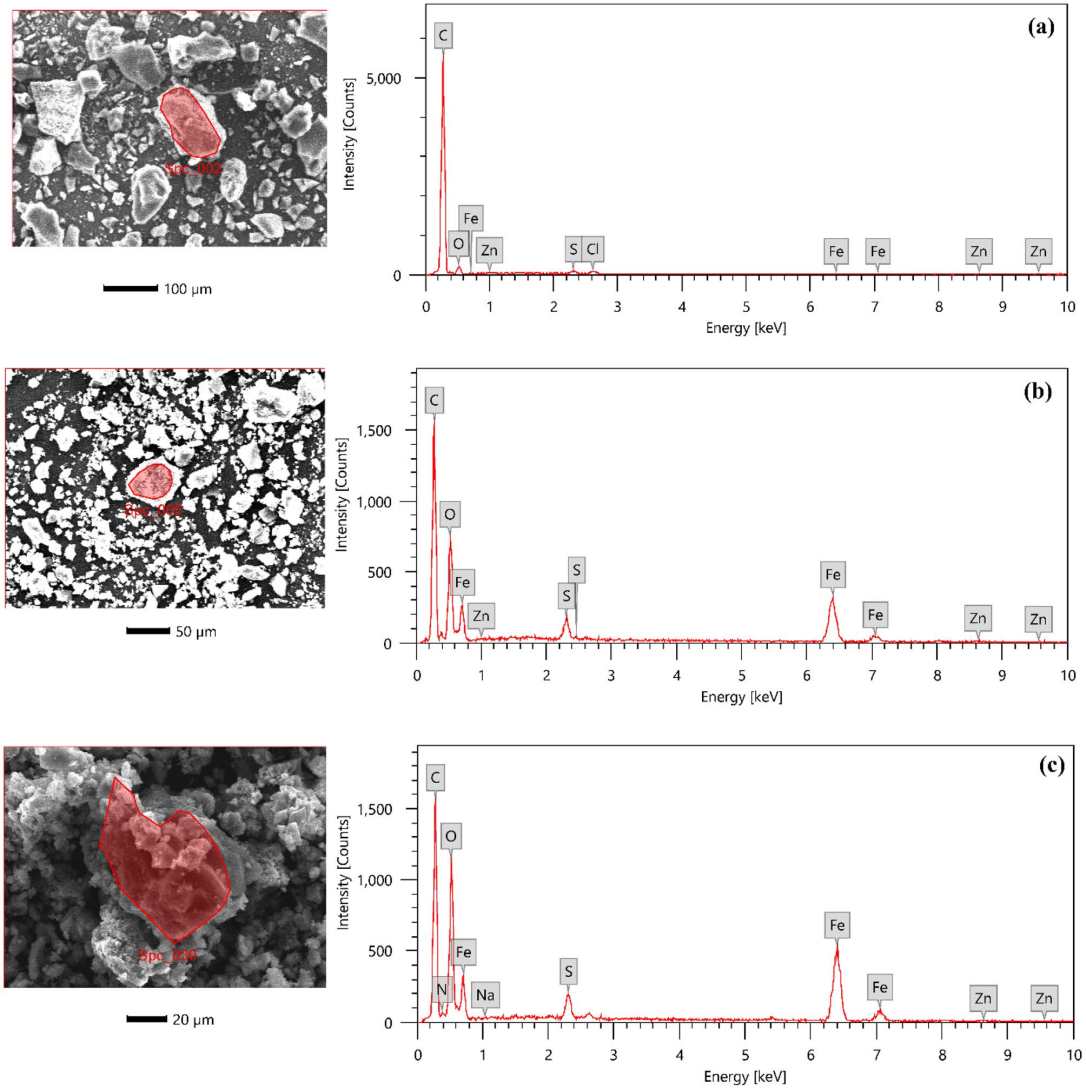
EDX spectrum for (**a**) OPAC, (**b**) MG-OPAC and (**c**) AO7 dye adsorbed on MG-OPAC surface.

The specific components that were on the surface of the samples under study were identified using energy-dispersive X-ray analysis (EDX). The EDX spectrum for activated carbon, which is a combination of OPAC and Fe_3_O_4_ (MG-OPAC) and AO7 dye adsorbed on MG-OPAC, is shown in Fig. 6. Figure 6a's OPAC (at 700 °C) spectrum reveals that the carbon concentration was highest, at 86.30%, while the oxygen content was second (11.99%). The usage of ZnCl_2_ during the chemical activation account for the presence of chlorine and zinc in the sample. The existence of five primary elements, namely carbon, oxygen, sulphur, zinc, and iron, is shown by the MG-OPAC (at 700 °C) spectrum in Fig. 6b. The presence of sulphur was attributed to the utilization of ferrous sulphate during the synthesis of MG-OPAC. While the iron was thought to have come from iron oxide that had precipitated on the surface of the activated carbon during the magnetization. The presence of N and S elements in Fig. 6c shows that AO7 aye adsorbed on the surface of magnetic activated carbon. Table 2 displays how the various components are composed on the various materials.

**Table 2:** Element composition on the different adsorbent materials.

Element	OPAC		MG-OPAC		AO7@MG-OPAC	
	Mass (%)	Atomic (%)	Mass (%)	Atomic (%)	Mass (%)	Atomic (%)
C	86.56	90.16	51.88	64.67	44.31	57.94
O	11.99	9.37	33.17	31.04	34.77	34.13
N	–	–	–	–	2.04	2.29
S	0.55	0.22	1.54	0.72	1.59	0.78
Na	–	–	–	–	0.05	0.03
Cl	0.49	0.17	–	–	–	–
Fe	0.07	0.02	12.70	3.40	16.75	4.71
Zn	0.35	0.07	0.72	0.16	0.49	0.12

### Adsorption study

#### Effect of solution pH on removal efficiency

**Figure 7:**
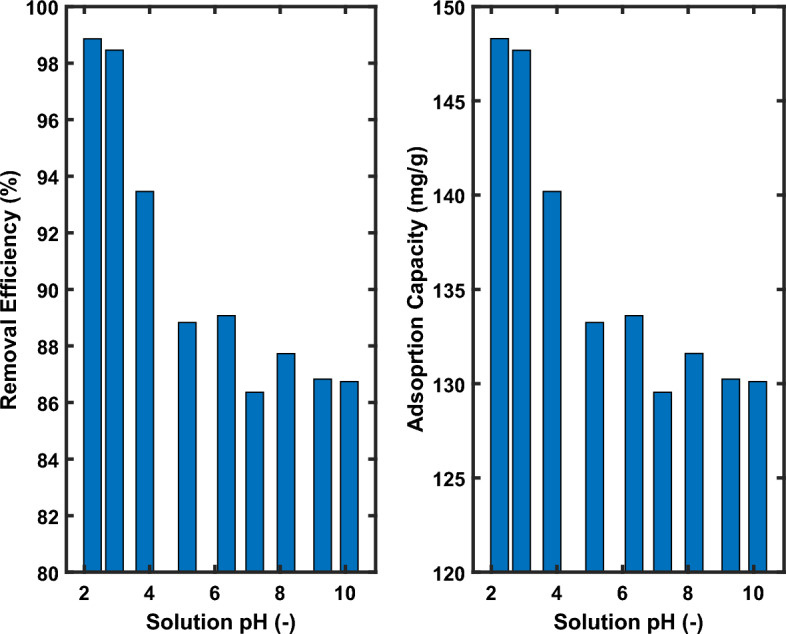
Effect of pH on the adsorption of AO7 dye on MG-OPAC [150 mg/L of AO.7 dye, 0.1 g/100 mL of MG-OPAC, 25 °C, 200 rpm, 180 min].

To look at how the pH of the solution affects the adsorption of the AO7 dye. All other parameters (AO7 dye concentration = 150 mg/L, stirring speed = 200 rpm, adsorbent dosage = 1.0 g/L, temperature = 25 °C) were held constant during the adsorption tests, which were conducted in the pH range of 2 to 10. The outcomes of the impact of solution pH are displayed in Fig. 7.

When we could see, when the pH of the solution increased, the greatest amount of AO7 dye could be absorbed at a pH of 2.25. Therefore, (pH 2.25) was chosen as the ideal pH level for additional adsorption trials since the adsorption capacity dropped. Higher adsorption at lower pH levels is brought on by an increase in H^+^ ions on the adsorbent surface, which causes electrostatic attraction between the positively charged adsorbent surface and the negatively charged anions of the dye (dye occurs in solution as negatively charged sulfonate anion) to be noticeably strong. The dual rivalry of both anions (C_1_6H_11_N_2_SO_4_^–^ and OH^–^) to be adsorbed on the surface of the adsorbent may be the cause of the reduction in the adsorption capacity at alkaline pH levels^11,61–63^.

#### Impact of MG-OPAC dose and contact time

**Figure 8:**
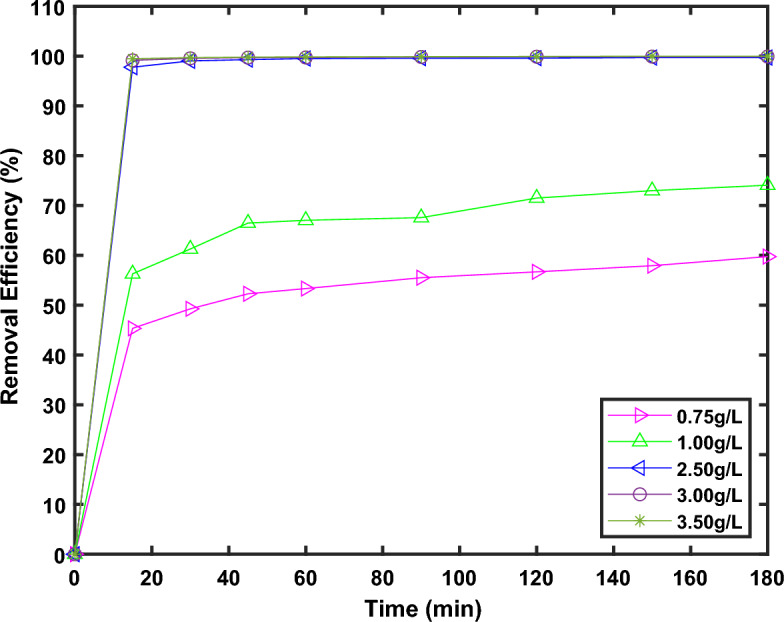
Contact time impact on the adsorption of AO7 dye at various dosage (0.75, 1.0, 2.53 and 3.5 g/L) of MG-OPAC using solutions with 500 mg/L primary concentration of AO7 dye at 200 rpm, 25 °C and 2.25 pH.

**Figure 9:**
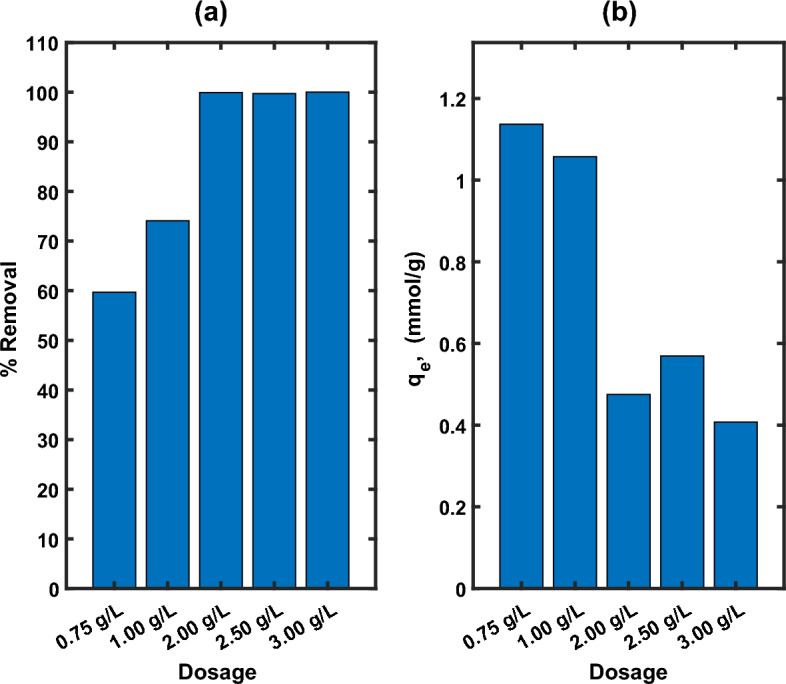
Effect of dosage on the maximum removal efficiency and *q*_e_ for AO7 dye initial concentration of 500 ppm.

The impact of MG-OPAC dose on the adsorption of AO7 dye kinetics was conducted over a 3-h period using solutions with a primary concentration of 500 mg/L adjusted to a pH of 2.25 at a temperature of 25 °C. The results are presented in Fig. 8. In all the 5 cases of dosage at least 40% removal was achieved within the first 15 min of contact time, which shows availability of abundance of active sites for adsorption at the beginning of the process. The maximum removal increased with increasing levels of dosage, which is expected since as more adsorbent material is added more active site are available for the removal of the adsorbate^64^. The maximum removal and amount of dye in solid phase obtained for the different dosages after three hours of contact time is reported in Fig. 9a,b, respectively.

#### Effect of dye initial concentration

**Figure 10:**
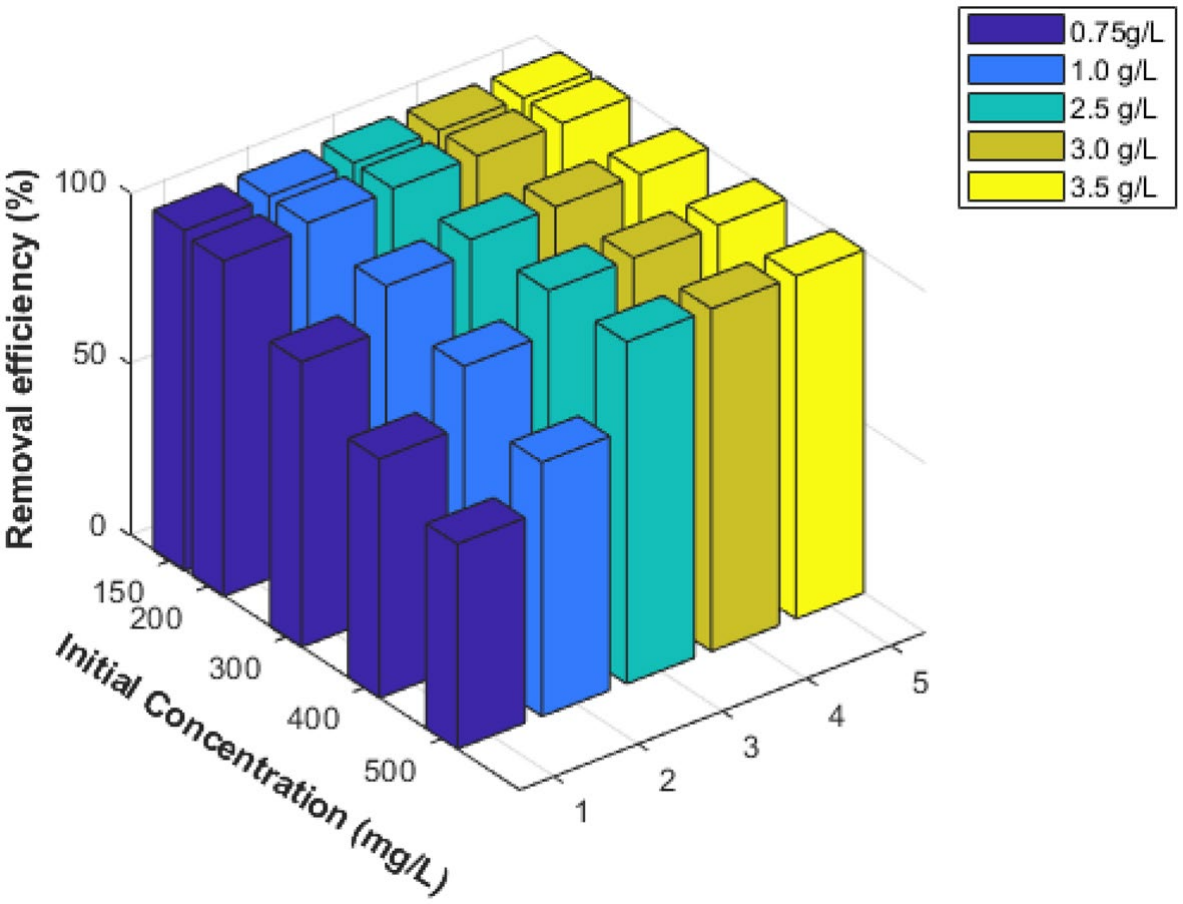
Effect of AO7 dye on the adsorption process.

**Figure 11:**
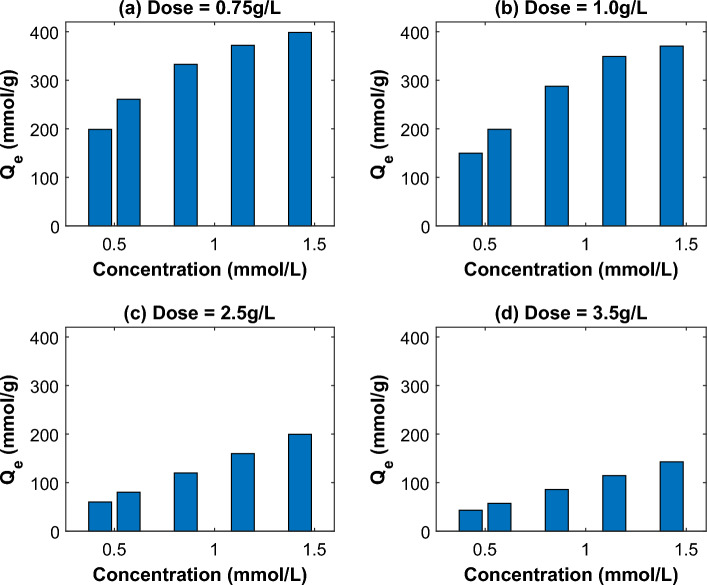
Use of various dosages of MG-OPAC to determine the relationship between the quantities of AO7 dye adsorbed at equilibrium (*q*_e_) and its starting concentration.

It was investigated how the initial concentration of AO7 dye in solutions influenced the rate of AO7 dye adsorption on MG-OPAC. The experiment was run for 3 h at various starting AO7 dye concentrations (150, 200, 300, 400, and 500 mg/L) and at varying adsorbent dosages (0.75–3.5 g/L) in the test solution at a temperature of 25 ± 2 °C and pH 2.25 (Fig. 10). The findings show that, for any given dosage of adsorbent, the percent of adsorption dropped as the initial concentration of the AO7 dye rose (this may be because the mass transfer driving force increased). However, as shown in Fig. 11a–d, when the concentration of AO7 dye in the test solution increased, so did the actual quantity of AO7 dye adsorbed per unit mass (adsorption capacity *q*_e_) of the adsorbent. This agrees with other results available in literature^26,33,48^.

### Modelling of the adsorption process

#### Adsorption isotherm study

The equilibrium studies' physicochemical information is adequate for characterizing the adsorption process as a singular operation, holding significant value in refining the design parameters of adsorption systems. Analyzing how solutes are distributed between the liquid and solid adsorbent phases serves as an indicator of the equilibrium state. To select a suitable model for incorporation into the design phase, it's imperative that the equilibrium data align accurately with various isotherm models^45^. Information on sorption processes, surface characteristics, and affinities of sorbents may be learned from the parameters obtained from the different isotherm models. Langmuir^65^, Freundlich^66^, Dubinin-Radushkevich^67^, Temkin^45^, Redlich-Peterson^45^, and generalised isotherm equations^45^ are some of the equations that may be used to represent equilibrium data. The applicability of isotherm models is evaluated by evaluating the correlation coefficients^45,65^.

The foundational assumption of the Langmuir adsorption process is the formation of a mono-layer of adsorbate molecules of ions on the outside of the adsorbent (in the absence of contact between adsorbed molecules), and that no more adsorption occurs (just a single adsorption layer) after that^65^. Furthermore, there is no adsorbate transmigration and uniform surface adsorption energies are assumed in this isotherm model. The Langmuir isotherm model was employed to determine the maximum adsorption capacity, which corresponds to the complete coverage of the sorbent surface by a monolayer. Equation (3) depicts the Langmuir nonlinear equation.

3 $${q}_{e}=\frac{{Q}_{m}{K}_{a}{C}_{e}}{1+{K}_{a}{C}_{e}}$$

${Q}_{m}$ (mg/g) is the maximum AO7 dye ions uptake per unit mass of MG-OPAC, which is correlated to the adsorption capacity and ${K}_{a} ({\text{L}}/{\text{mol}})$ is Langmuir constant that is exponentially proportional to the heat of adsorption and related to the intensity of adsorption.

The initial and foundational framework for understanding the sorption process is provided by the Freundlich isotherm model^66^. Furthermore, employing the Freundlich equation presupposes that the sorption energy undergoes exponential reduction as the sorption sites on an adsorbent become fully saturated. This conceptual framework is well-suited for describing adsorption on surfaces characterized by heterogeneity, where interactions take place among the adsorbed molecules. Equation (4) symbolizes the non-linear form of the Freundlich equation."

4 $${q}_{e}={K}_{F}{C}_{e}^\frac{1}{n}$$

In Eq. (4), ${K}_{F}$ (L/mg) is the Freundlich adsorption constant indicative of the relative adsorption capacity of the adsorbent, and $1/n$ is a constant indicating the adsorption intensity of sorbate onto the sorbent or surface heterogeneity. The adsorbent surface which is more heterogeneous is indiacted by low values of $1/n$ . A value for $1/n$ below 1 indicates a normal Langmuir isotherm, while 1/*n* above 1 is indicative of cooperative adsorption^45,67^.

According to the Temkin isotherm model, all, the molecules in the layer's heat of adsorption will drop linearly with coverage as a result of adsorbate-adsorbent interactions. Additionally, the adsorption process will be distinguished by a uniform distribution of the highest binding energy. In contrast to what the Freundlich equation predicts, the Temkin isotherm's derivation assumes that the decline in the heat of sorption is linear^67^. The Temkin equation is written as Eq. (5):

5 $${q}_{e}=Bln\left(A{C}_{e}\right)$$

The constant, *B* can be written as *B* =$\frac{RT}{b}$ *where b* related to the heat of adsorption. The Temkin equilibrium binding constant, A (L/mg), corresponds to the highest binding energy.

As an empirical model, the Dubinin-Radushkevich isotherm is used to represent the adsorption of subcritical vapours onto micropore materials using a pore filling process^67^. Equation (6) describes the Dubinin-Radushkevich isotherm model^45,67^:

6 $${q}_{e}={q}_{DR}{\text{exp}}(-{K}_{DR}{\varepsilon }^{2})$$

The parameter ε is calculated as Eq. (7):

7 $$\varepsilon =RT{\text{ln}}\left[1+\frac{1}{{C}_{e}}\right]$$

Within Eqs. (6, 7), ${q}_{DR}$ represents the maximum adsorption capacity of AO7 dye in units of mg/g. *K*_DR_ denotes the Dubinin–Radushkevich constant, while R signifies the universal gas constant. T stands for the absolute temperature measured in Kelvin. The connection between *K*_DR_ and the average free energy of biosorption (*E*_DR_) exists as the alteration in free energy for the migration of one mole of adsorbate ion from an infinite distance in the solution to the adsorbent surface. The apparent energy *E*_*DR*_ can be calculated as Eq. (8):

8 $${E}_{DR}=\frac{1}{\sqrt{2{K}_{DR}}}$$

The Redlich-Peterson isotherm is expressed as Eq. (9):

9 $${q}_{e}=\frac{{q}_{max}{B}_{pb}{C}_{e}}{1+{B}_{pb}{C}_{e}^{\beta }}$$

In Eq. (9) *B*_*pb*_ is the Redlich-Peterson isotherm constant (L/mmol); ${q}_{max}$ is the maximum loading achieved and *ß* is an exponent that lies between 0 and 1.

**Figure 12:**
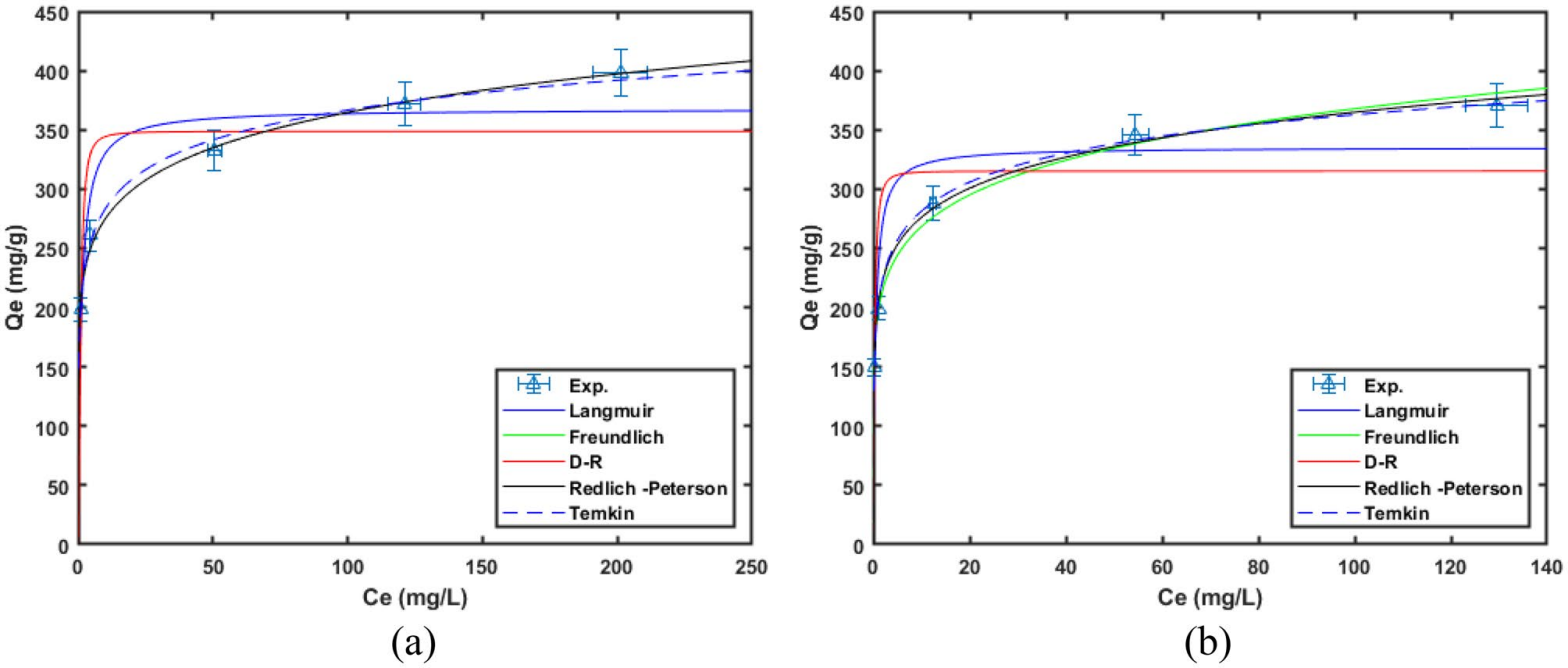
Comparison of different isotherm models at different doses of MG-OPAC adsorbent (**a**) 0.75 g/L (**b**) 1.00 g/L. All experiments performed with solutions with adjusted pH of 2.25.

A bespoke written MATLAB code that utilises non-linear least-square regression fitting was used to fit the model given be Eqs. (3), (4), (5), (7) and (9) to the experimental data obtained at adsorbent dose of 0.75 g/L and 1.0 g/L. The comparison of the fits of these models to the experimental data is shown in Fig. 12. The summary of the model parameters is given in Table 3. From Fig. 12 and Table 3, Temkin model shows the best fit model within the applied isotherm models.

**Table 3:** Summary of the isotherm model parameters obtained from fitting to experimental data obtained at different adsorbent dose level.

Isotherm model	Parameters	MG-OPAC dose (g/L)	
		0.75	1.0
Langmuir	*K*_*a*_	0.87	2.17
	*Q*_*m*_ (mg/g)	367.71	335.22
	*R*^*2*^	0.841	0.771
	*RSME*	32.7580	45.1460
Freundlich	*K*_*F*_	206.710	195.520
	*n*	8.112	7.290
	*R*^*2*^	0.986	0.986
	*RSME*	9.567	11.080
Dubinin	*K*	0.000	0.000
	*q*	348.670	315.120
	*R*^*2*^	0.608	0.564
	*RSME*	51.354	62.299
Redlich-Peterson	*B*_*pb*_	7835.814	19.539
	*a*	0.877	0.881
	*q*_*m*_* (mg/g)*	206.711	211.395
	*R*^*2*^	0.980	0.992
	*RSME*	11.711	8.246
Temkin	*A*	241.37	280.52
	*B*	36.330	35.410
	*R*^*2*^	0.990	0.997
	*RSME*	8.175	4.791

As can be seen Table 3 the Langmuir, Freundlich, Temkin and the Redlich-Peterson models all have high *R*^2^ values of greater than 0.8 indicating their suitability for description of the experimental data. The MG-OPAC has a Langmuir adsorption capacity of about 367 mg/L. the Langmuir capacity of the MG-OPAC is comparable to other values reported in literature^34–36,62,68,69^. Given that the Temkin model has the lowest residual error value, it is the most effective one for explaining the experimental data. It follows that the heat of adsorption falls linearly with coverage and that the binding energies are distributed uniformly since the Temkin model is the best one for characterising the data.

Table 3 makes it clear that (1/*n*) values were less than one, indicating that the surface nature of the MG-OPAC is heterogeneous and favourable for the adsorption process. The '*n*' value is larger than 1, suggesting a favourable physical mechanism for the adsorption of AO7 dye onto MG-OPAC^68^.

#### Kinetic studies of adsorption

Different kinetic models were fitted to the experimental data to get in insight to the adsorption mechanism, which governs the adsorption process^69–75^. In this study, the adsorption of the AO7 dye was examined using the kinetic models of Elovich^69–71^, pseudo-1st order^72^, and pseudo-2nd order^73^, as well as film diffusion^74^ and intraparticle diffusion^75^. The correlation coefficients (*R*^2^) were used to represent how well the experimental data and model-predicted values matched. The following Eq. (10, 11)^72^ often expresses the pseudo-1st order kinetic model.

10 $$\frac{d{q}_{t}}{dt}={k}_{1}\left({q}_{e}-{q}_{t}\right),$$

where *q*_*t*_ is the amount of solute adsorbed at time *t* (min), *q*_*e*_ is the amount of solute (mg g^−1^) adsorbed at saturation and* k*_*1*_ is the pseudo-first-order rate constant (min^−1^). The integrated form of the Eq. (10) is expressed as Eq. (11):

11 $${q}_{t}={q}_{e}\left(1-exp\left(-{k}_{1}t\right)\right)$$

The values of *q*_e_ and *k*_*1*_ was calculated from the kinetics data through non-linear regression of the kinetics data. The model parameters are summarised in Table 4. It is clear from the findings that the pseudo-2nd order model, as opposed to the pseudo-1st -order model, was fitted with high linearity by *R*^2^ (> 0.999).

**Table 4:** Summary of the parameters obtained from non-linear fit of Pseudo-First-Order kinetic model to the experimental data obtained from different conditions of adsorbent dosage and initial AO7 dye concentrations.

Dose g/L	Parameter	AO7 dye initial concentration of solution (mg/L)				
		100	200	300	400	500
0.75	*k*	0.1826 (0.1497, 0.2154)	0.146 (0.1201, 0.1719)	0.1084 (0.07448, 0.1424)	0.1128 (0.07261, 0.153)	0.09911 (0.06415, 0.1341)
	*q*_*max*_	195.7 (193.5, 198)	256.2 (252.1, 260.3)	317.2 (304.7, 329.7)	352 (336.8, 367.2)	371.7 (353.8, 389.6)
	sse	42.3497	136.6543	1181.2229	1766.1213	2350.332
	rmse	2.4597	4.4184	12.9902	15.8841	18.324
	*R*^2^	0.9986	0.9976	0.9866	0.9837	0.981
	adj *R*^2^	0.9986	0.9973	0.9847	0.9814	0.978
1.0	*k*	0.2629 (0.2267, 0.2992)	0.2666 (0.2161, 0.317)	0.116 (0.08916, 0.1429)	0.09374 (0.06526, 0.1222)	0.09756 (0.06433, 0.1308)
	*q*_*max*_	149 (148.5, 149.6)	197.5 (196.6, 198.5)	278.5 (270.9, 286.1)	328.7 (314.4, 343)	348.6 (332.2, 365.1)
	sse	2.6853	8.1884	445.8286	1468.0642	1969.8000
	rmse	0.6194	1.0816	7.9806	14.4818	16.7752
	*R*^2^	0.9999	0.9998	0.9934	0.9844	0.9815
	adj *R*^2^	0.9998	0.9997	0.9925	0.9822	0.9789
2.5	*k*	0.4087 (0.3347, 0.4826)	0.3787 (0.3376, 0.4198)	0.3938 (0.3362, 0.4514)	0.3861 (0.3252, 0.4471)	0.3137 (0.272, 0.3554)
	*q*_*max*_	59.94 (59.89, 59.99)	79.88 (79.82, 79.94)	119.8 (119.7, 119.9)	159.5 (159.3, 159.7)	199 (198.6, 199.4)
	sse	0.0229	0.0309	0.0866	0.2166	1.3859
	rmse	0.0573	0.0664	0.1112	0.1759	0.4449
	*R*^2^	1.0000	1.0000	1.0000	1.0000	1.0000
	adj *R*^2^	1.0000	1.0000	1.0000	1.0000	1.0000
3.0	*k*	0.416 (0.374, 0.4581)	0.4101 (0.3526, 0.4677)	0.4466 (0.3834, 0.5098)	0.3905 (0.3391, 0.442)	0.3368 (0.3044, 0.3692)
	*q*_*max*_	49.98 (49.96, 50)	66.63 (66.59, 66.67)	99.89 (99.85, 99.93)	133.1 (133, 133.2)	166.3 (166.1, 166.5)
	sse	0.00410	0.01640	0.01490	0.09420	0.29230
	rmse	0.02430	0.04850	0.04620	0.11600	0.20440
	*R*^2^	1.00000	1.00000	1.00000	1.00000	1.00000
	adj *R*^2^	1.00000	1.00000	1.00000	1.00000	1.00000
3.5	*k*	0.4811 (0.3843, 0.5778)	0.4525 (0.4005, 0.5046)	0.4437 (0.3764, 0.511)	0.4415 (0.3655, 0.5176)	0.398 (0.3363, 0.4597)
	*q*_*max*_	42.84 (42.83, 42.86)	57.12 (57.11, 57.14)	85.66 (85.62, 85.7)	114.2 (114.1, 114.2)	142.7 (142.5, 142.8)
	sse	0.0023	0.0028	0.0136	0.0328	0.1247
	rmse	0.0181	0.0199	0.0440	0.0685	0.1335
	*R*^2^	1.0000	1.0000	1.0000	1.0000	1.0000
	adj *R*^2^	1.0000	1.0000	1.0000	1.0000	1.0000

The pseudo -2nd order kinetic model^73^ is generally expressed as Eq. (12):

12 $${q}_{t}=\frac{{k}_{2}{q}_{e}^{2}t}{1+{k}_{2}{q}_{e}t},$$

where *q*_e_ is the amount adsorbed at equilibrium (mg/g) and *K*_2_ (g min^-1^ mg^−1^) is the pseudo-second-order rate constant. The kinetics adsorption behaviour of AO7 dye on MG-OPAC is depicted in .

**Figure 13:**
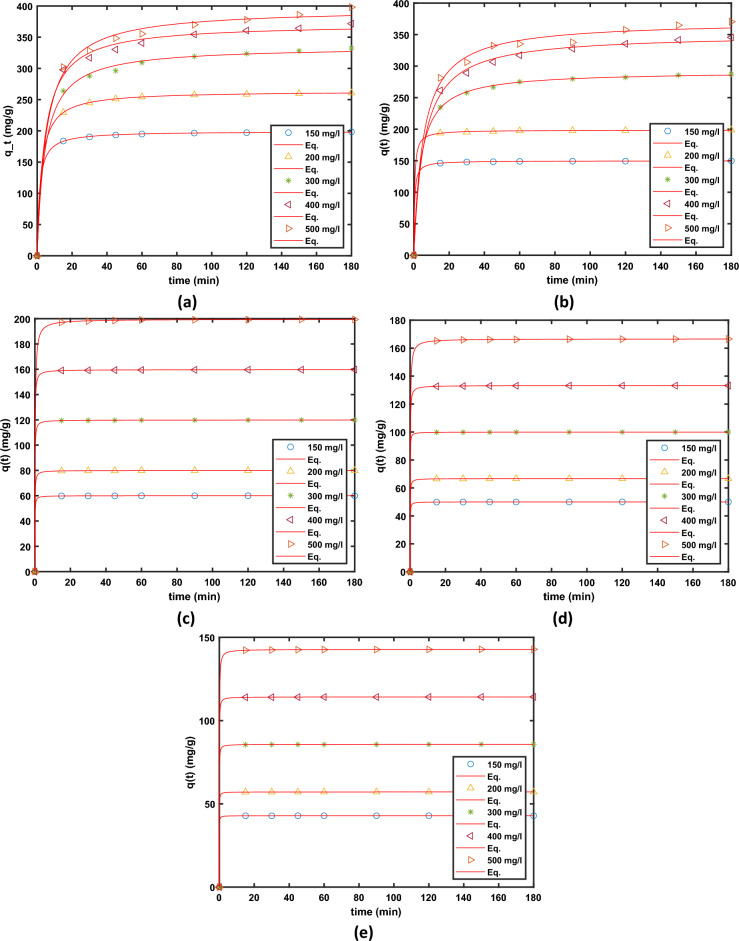
Fit of the Pseudo-2nd-order models to the adsorption kinetic data obtained and different dosages and different initial concentration of AO7 dye (**a**) 0.75 g/L, (**b**) 1.0 g/L (**c**) 3.0 g/L, (**d**) 3.0 g/L, and (**e**) 3.5 g/L of MG-OPAC.

Table 5 and Fig. 13a–e. It is clear from the findings that the pseudo-second-order model, as opposed to the pseudo-first-order model, was fitted with high linearity by *R*^2^ (> 0.999). It should be highlighted that a pseudo-second-order kinetic model has greater agreement between the experimental and estimated *q*_e_ values than a pseudo-first-order kinetic model.

**Table 5:** Summary of the parameters obtained from non-linear fit of Pseudo–2nd Order kinetic model to the experimental data obtained from different conditions of adsorbent dosage and initial AO7 dye concentrations.

Dose	*Parameter*	AO7 dye initial concentration of solution (mg/L)				
g/L		100	200	300	400	500
3.5	*k*_*2*_	1.158 (0.4773, 1.838)	0.708 (0.5221, 0.8939)	0.3774 (0.2382, 0.5165)	0.266 (0.1416, 0.3904)	0.1174 (0.07948, 0.1553)
	*q*_*e*_	42.86 (42.84, 42.87)	57.15 (57.14, 57.16)	85.7 (85.67, 85.73)	114.2 (114.2, 114.3)	142.8 (142.7, 142.9)
	*sse*	0.0009	0.0005	0.0035	0.0113	0.0276
	*rmse*	0.0116	0.0085	0.0224	0.0403	0.0627
	*R*^*2*^	1.0000	1.0000	1.0000	1.0000	1.0000
	*adj R*^*2*^	1.0000	1.0000	1.0000	1.0000	1.0000
3.0	*k*_*2*_	0.4937 (0.4202, 0.5673)	0.3115 (0.2136, 0.4094)	0.3488 (0.2228, 0.4749)	0.119 (0.09227, 0.1456)	0.0479 (0.04436, 0.05143)
	*q*_*e*_	50.01 (50, 50.02)	66.68 (66.65, 66.71)	99.94 (99.91, 99.97)	133.2 (133.2, 133.3	166.6 (166.6, 166.7)
	*sse*	0.0003	0.0037	0.0039	0.0129	0.0085
	*rmse*	0.0069	0.0231	0.0237	0.0430	0.0348
	*R*^*2*^	1.0000	1.0000	1.0000	1.0000	1.0000
	*adj R*^*2*^	1.0000	1.0000	1.0000	1.0000	1.0000
2.5	*k*_*2*_	0.3111 (0.1726, 0.4497)	0.1786 (0.1462, 0.211)	0.1343 (0.09602, 0.1725)	0.08831 (0.06011, 0.1165)	0.02661 (0.02252, 0.0307)
	*q*_*e*_	59.99 (59.95, 60.03)	79.97 (79.94, 80)	119.9 (119.8, 119.9)	159.7 (159.6, 159.8)	199.6 (199.4, 199.7)
	*sse*	0.0075	0.0038	0.0164	0.0475	0.1170
	*rmse*	0.0327	0.0231	0.0484	0.0824	0.1293
	*R*^*2*^	1.0000	1.0000	1.0000	1.0000	1.0000
	*adj R*^*2*^	1.0000	1.0000	1.0000	1.0000	1.0000
1.0	*k*_*2*_	0.01699 (0.01518, 0.0188)	0.0124 (0.00934, 0.0155)	0.000912 (0.000822, 0.0010)	0.000506 (0.000402, 0.000609)	0.000502 (0.000333, 0.00067)
	*q*_*e*_	149.9 (149.7, 150.1)	198.8 (198.2, 199.3)	291.7 (289.5, 294)	350.6 (343.3, 358)	371.3 (359, 383.6)
	*sse*	0.13070	1.32610	17.36780	164.85623	470.78470
	*rmse*	0.13665	0.43525	1.57516	4.85293	8.20091
	*R*^*2*^	1.00000	1.00000	0.99974	0.99825	0.99558
	*adj R*^*2*^	1.00000	1.00000	0.99971	0.99800	0.99495
0.75	*k*_*2*_	0.00379 (0.00348, 0.0041)	0.00167 (0.00163, 0.00172)	0.000665 (0.0005, 0.00083)	0.000623 (0.000421, 0.000825)	0.000475 (0.000324, 0.000626)
	*q*_*e*_	199.4 (198.9, 200)	263.9 (263.5, 264.3)	335.2 (328.1, 342.3)	371.7 (361.4, 381.9)	396 (383.6, 408.3)
	*sse*	1.2341	0.5597	168.0976	351.2968	478.9685
	*rmse*	0.4199	0.2828	4.9004	7.0842	8.2719
	*R*^*2*^	1.0000	1.0000	0.9981	0.9968	0.9960
	*adj R*^*2*^	1.0000	1.0000	0.9978	0.9963	0.9955

**Figure 14:**
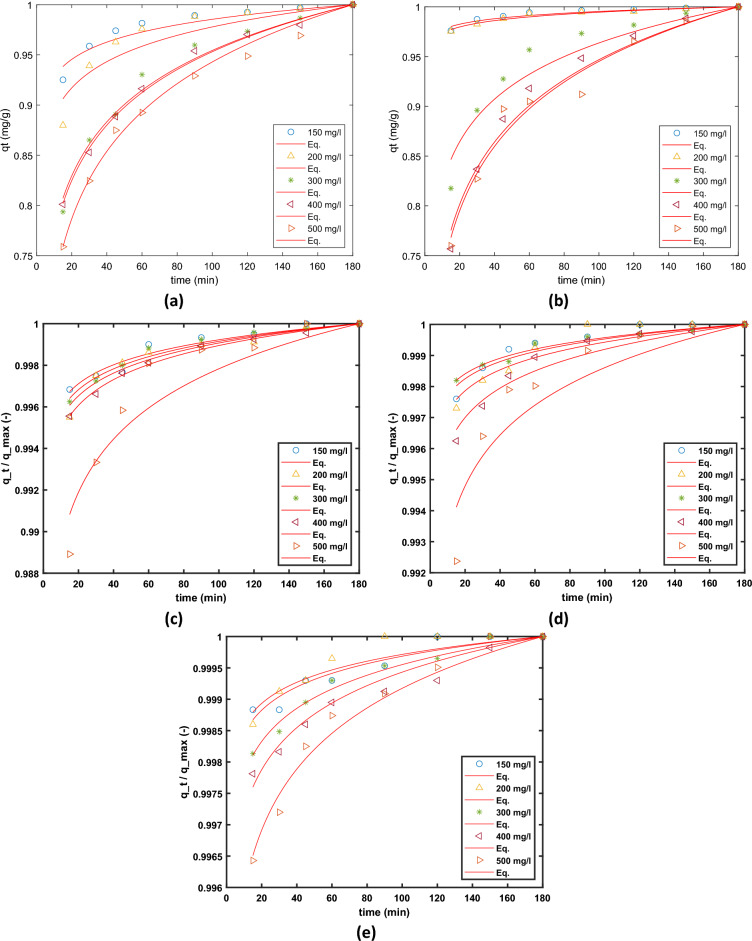
Fit of the Elovich model to the adsorption kinetic data obtained and different dosages and different initial concentration of AO7 dye (**a**) 0.75 g/L, (**b**) 1.0 g/L (**c**) 3.0 g/L, (**d**) 3.0 g/L, and (**e**) 3.5 g/L of MG-OPAC.

The Elovich model Eq. (13) describes the chemisorption behaviour between adsorbent and adsorbate^69–71^. The adsorption capacity typically provided by the Eqs. (10), (11) is the basis for the Elovich equation, which is a rate equation.

13 $${q}_{t}={q}_{ref}\left(1+{R}_{E} ln(\frac{t}{{t}_{ref}})\right),$$

where ${q}_{ref}$ is the maximum concentration of the adsorbate in solid phase achieved after a time ${t}_{ref}$, the parameter ${R}_{E}$ is given as ${R}_{E}=\frac{1}{{q}_{ref}b}$. The non-linear regression fits of Elovich model to the experimental data for different doses of adsorbent are presented in Fig. 14.

**Figure 15:**
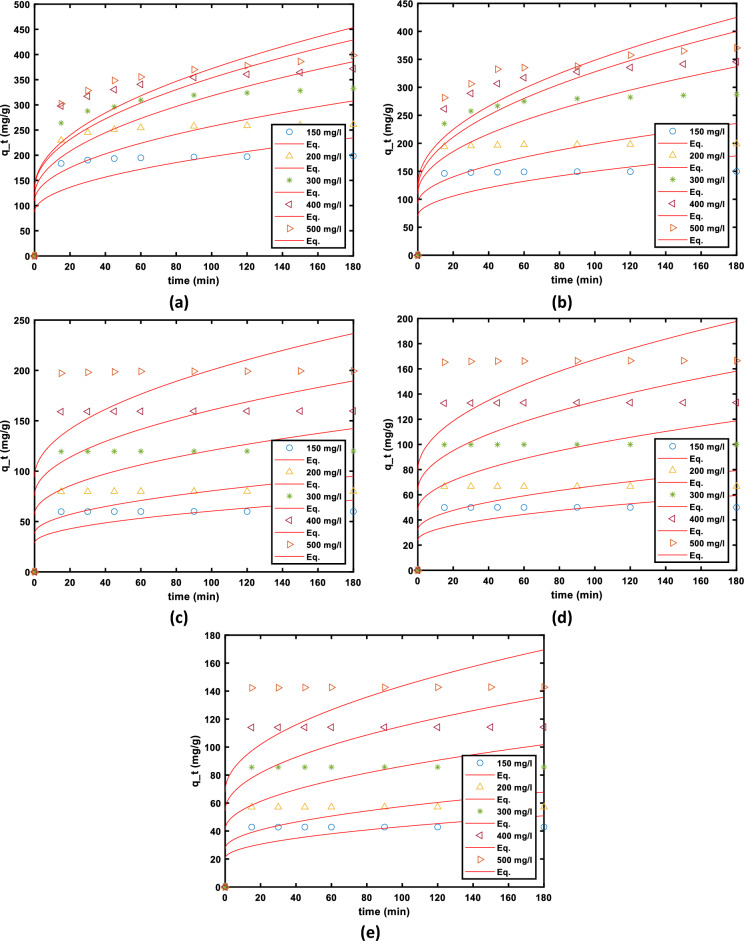
Fit of the Intra-particle diffusion model to the adsorption kinetic data obtained and different dosages and different initial concentration of AO7 dye (**a**) 0.75 g/L, (**b**) 1.0 g/L (**c**) 3.0 g/L, (**d**) 3.0 g/L, and (**e**) 3.5 g/L of MG-OPAC.

The adsorption process, which occurs through multiple stages, encompasses the movement (transfer) of adsorbate molecules (AO7) from the liquid phase to the particles of the solid phase (MG-OPAC). This is succeeded by the subsequent diffusion of the solute molecules into the internal pores. In cases of batch systems with rapid agitation, intraparticle diffusion might emerge as the controlling factor dictating the progression of the experiment^75^. Intraparticle diffusion is another kinetic model that ought to be utilised to investigate the rate-limiting phase for AO7 dye adsorption onto MG-OPAC since it is likely that AO7 dye is transferred from its aqueous solution to MG-OPAC via intraparticle diffusion. Equation (14) may be used to express the intra-particle diffusion.

14 $${q}_{t}={K}_{dif}{t}^\frac{1}{2}+C$$

where *K*_dif_ is the intra-particle diffusion rate constant (mg/g). The values of *C* (intercept) provide information about the size of the boundary layer and resistance to mass transfer. The resistance to the external mass transfer increases as the intercept increases, see Table 6. The non-linear regression fits of the model are presented in Fig. 15.

**Table 6:** Summary of the parameters obtained from non-linear fit of Intraparticle Diffusion model to the experimental data obtained from different conditions of adsorbent dosage and initial AO7 dye concentrations.

Dose g/L	Parameter	Initial concentration of solution				
		100 mg/L	200 mg/L	300 mg/L	400 mg/L	500 mg/L
0.75	*b*	87.21 (5.735, 168.7)	109 (6.914, 211.1)	119 (7.377, 230.7)	132.5 (8.479, 256.5)	132.8 (8.348, 257.3)
	*RE*	0.02481 (0.02, 0.02963)	0.03775 (0.02809, 0.0474)	0.07761 (0.07133, 0.08389)	0.07976 (0.07665, 0.08286)	0.09711 (0.09208, 0.1021)
	*sse*	0.00038	0.0015	0.00065	0.000159	0.000417
	*rmse*	0.0074	0.0148	0.0096	0.0048	0.0077
	*R*^*2*^	0.9125	0.8658	0.9814	0.9952	0.99060
	*adj R*^*2*^	0.9125	0.8658	0.9814	0.9952	0.99060
1.0	*b*	69.77 (4.644, 134.9)	92.31 (6.146, 178.5)	109.5 (6.605, 212.3)	116.6 (6.867, 226.3)	124.6 (7.448, 241.7)
	*RE*	0.00796 (0.006487, 0.009435)	0.0092 (0.00816, 0.0103)	0.06175 (0.05077, 0.07274)	0.0904 (0.08258, 0.09823)	0.09331 (0.08403, 0.1026)
	*sse*	0.0000359	0.000019	0.00199	0.00101	0.00142
	*rmse*	0.00226	0.00164	0.017	0.012	0.014
	*R*^*2*^	0.9179	0.9617	0.9250	0.9793	0.9692
	*adj R*^*2*^	0.9179	0.9617	0.9250	0.9793	0.9692
2.5	*b*	28.58 (1.904, 55.25)	38.06 (2.536, 73.58)	57.07 (3.803, 110.3)	75.97 (5.062, 146.9)	94.2 (6.275, 182.1)
	*RE*	0.00132 (0.00112, 0.00151)	0.00158 (0.00137, 0.00179)	0.00144 (0.001315, 0.00158)	0.00179 (0.00171, 0.00186)	0.00369 (0.002858, 0.00452)
	*sse*	0.000000608	0.0000008	0.0000003	0.0000	0.0000115
	*rmse*	0.0003	0.0003	0.00021	0.0001	0.0013
	*R*^*2*^	0.9421	0.9492	0.9742	0.9944	0.8911
	*adj R*^*2*^	0.9421	0.9492	0.9742	0.9944	0.8911
3.0	*b*	23.85 (1.589, 46.11)	31.78 (2.118, 61.45)	47.7 (3.178, 92.22)	63.43 (4.227, 122.6)	79.01 (5.264, 152.8)
	*Re*	0.000797 (0.000624, 0.00097)	0.00097 (0.000746, 0.0012)	0.000725 (0.000651, 0.0008)	0.00137 (0.00116, 0.00157)	0.00237 (0.00175, 0.00299)
	*sse*	0.00000049	0.00000085	0.00000009	0.00000066	0.00000636
	*rmse*	0.0003	0.0003	0.0001	0.0003	0.0010
	*R*^*2*^	0.9003	0.8889	0.9674	0.9464	0.8617
	*adj R*^*2*^	0.9003	0.8889	0.9674	0.9464	0.8617
3.5	*b*	20.46 (1.364, 39.57)	27.29 (1.818, 52.75)	40.9 (2.725, 79.07)	54.49 (3.631, 105.4)	67.99 (4.531, 131.5)
	*RE*	0.000535 (0.000439, 0.00063)	0.00049 (0.000367, 0.00060)	0.00076 (0.000698, 0.000825)	0.000966 (0.00086, 0.00108)	0.00141 (0.00127, 0.00154)
	*sse*	0.000000150	0.000000232	0.000000067	0.000000198	0.000000282
	*rmse*	0.0001	0.0002	0.0001	0.0002	0.0002
	*R*^*2*^	0.9126	0.8816	0.9796	0.9512	0.9761
	*adj R*^*2*^	0.9126	0.8816	0.9796	0.9512	0.9761

The information provided in Table 6 illustrates the presence of non-zero C values, indicating the presence of a certain extent of boundary layer influence. This observation underscores the possibility of additional mechanisms governing the adsorption rate apart from intraparticle diffusion, which conventionally holds the role of the limiting factor in the adsorption of AO7 dye onto MG-OPAC. The *R*^2^ values presented in the Table 6 are also very low, which indicates the model is not appropriate for describing the experimental data.

### Regeneration of MG-OPAC

**Figure 16:**
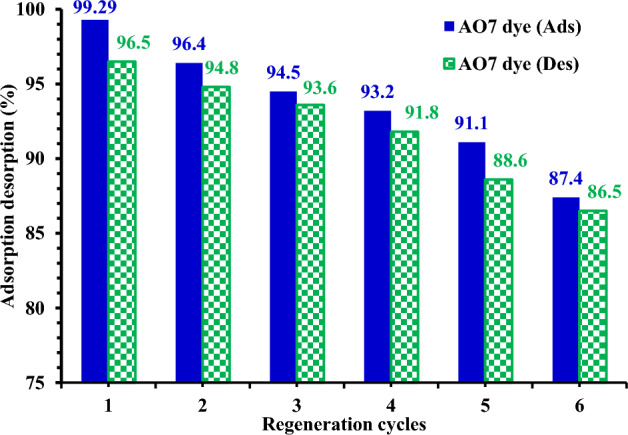
AO7 dye was desorption% from MG-OPAC (0.75 g/L) by 0.1 M NaOH and HCl, and MG-OPAC regeneration was used to promote AO7 dye (150 mg/L) adsorption cycles.

To test the viability and reusability of MG-OPAC as an adsorbent, desorption tests of the AO7 dye from the MG-OPAC adsorbent were carried out by 0.1 M NaOH and HCl as elution media. With increasing regeneration cycles in this situation, the desorption percentage dropped (Fig. 16). The regenerated MG-OPAC was used in six successive adsorption/desorption cycles. The amount of adsorption that was offered remained constant during the cycles; however, after six generations, the adsorption capacity had decreased by 11.89%, while the desorption capacity decreased by 10% after six desorption cycles. It implies that it might be employed as a long-lasting AO7 dye adsorption process (Fig. 16).

### Adsorption mechanism of AO7 dye ions by MG-OPAC

**Figure 17:**
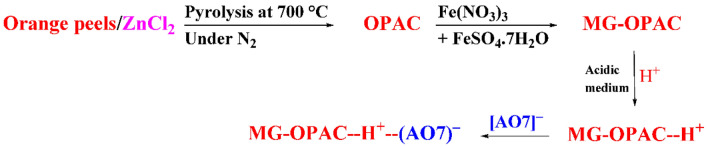
Probable mechanism for the AO7 dye ions adsorption onto the MG-OPAC in acidic medium.

In the case of Acid Orange 7 dye, the probable adsorption mechanism onto magnetic activated carbon (MG-OPAC) in acidic medium was explained in Fig. 17. The activated carbon possesses numerous surface functional groups such as hydroxyl (–OH), carboxyl (–COOH), and other polar moieties. These functional groups play a crucial role in attracting and holding the dye molecules. Acid Orange 7 dye is likely to have charged particles, as many dyes are ionic or polar in nature. The activated carbon, being a porous material, has a large surface area with a distribution of positive and negative sites. After the pyrolysis of the orange peels at 700 °C under nitrogen gas, many functional groups were formed on the adsorbent (OPAC) surface like allene C=C=C, ketamine C=C=N, hydroxyl O–H, and C-N groups. Activated carbon, with its graphitic structure, can form π-π interactions with these aromatic rings. This type of interaction enhances the adsorption capacity, especially when the activated carbon is magnetic. The mechanism of the removal of AO7 dye ions in an acidic medium may be achieved via physical interaction due to electrostatic interaction between the positive hydrogen ions in the bulk solution and the nitrogen and oxygen functional groups on the MG-OPAC surface, then surface charge became positive; subsequently electrostatic interaction was occurred between the positively charged surface and the predominant AO7 dye anionic species (AO7)^–^.

## Conclusion

The research centres on the effective removal of AO7 dye from wastewater utilizing magnetic activated carbon (MG-OPAC). Renowned for their robust adsorption capabilities, MG-OPAC offer the distinct advantage of facile separation from the medium through the application of an external magnetic field. Optimal conditions were determined through rigorous analysis, showcasing that the highest AO7 dye adsorption occurred at pH 2.25, with a contact time of 180 min (equivalent to 3 h). Remarkably, the adsorption percentage exhibited an upward trajectory in correlation with the adsorbent dose, culminating in maximum adsorption efficacy at a dose of 0.75 g/L as 357.14 mg/g. The maximum removal % was 99.29% using 150 mg/L of AO7 dye concentration and 0.75 g/L dose of MG-OPAC. While the percentage of adsorption declined as the AO7 dye concentration increased, the actual amount of AO7 dye adsorbed per unit mass (adsorption capacity *q*_e_) of the adsorbent experienced a discernible rise. The acquired experimental data were effectively elucidated by the Langmuir, Freundlich and Temkin isotherm models, offering comprehensive insights into the adsorption process. Furthermore, the kinetics data harmoniously aligned with the pseudo-second-order kinetics model with *R*^2^ very close to unity, affirming its reliability and suitability in depicting the experimental outcomes. The adsorption mechanism of AO7 dye using MG-OPAC was demonstrated based on the electrostatic attraction between the dye anion and the positively charged MG-OPAC in acidic medium. The regeneration of MG-OPAC was studied for six cycles and was found to be excellent for use as an adsorbent for treating AO7 dye present in textile industrial wastewater.

### Supplementary Information

Supplementary Figures.

## Data Availability

The datasets used in this investigation are accessible for review upon request from the corresponding author of the paper.
