# Supplementary material for: Orange peel magnetic activated carbon for removal of acid orange 7 dye from water
Source: Sci Rep. 2024 Jan 2;14:119. doi: 10.1038/s41598-023-50273-3 (PMC10761961; doi:10.1038/s41598-023-50273-3)
Supplement: Supplementary file 1 — Supplementary Figures. [file 41598_2023_50273_MOESM1_ESM.docx]

**Supplement data**

**Orange Peel Magnetic Activated Carbon for removal of Acid Orange 7 dye from water**

Asmaa Khalil, Chirangano Mangwandi, Mohamed A. Salem, Safaa Ragab, Ahmed El Nemr*


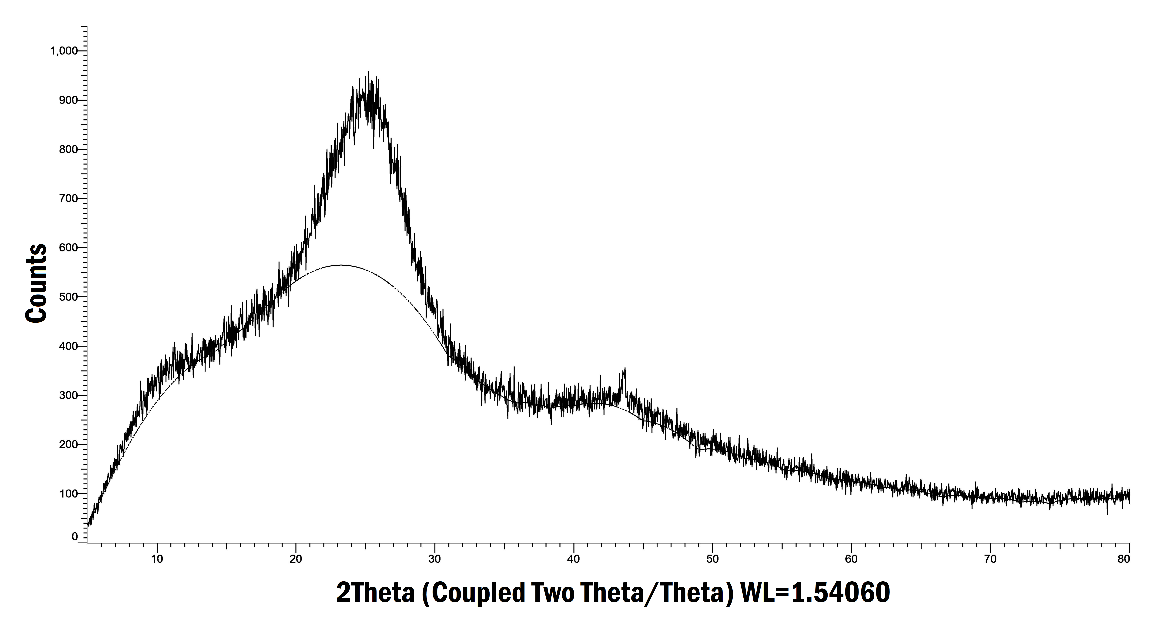


**Figure S1**. XRD analysis of OPAC prepared at 700 °C under N_2_.

| 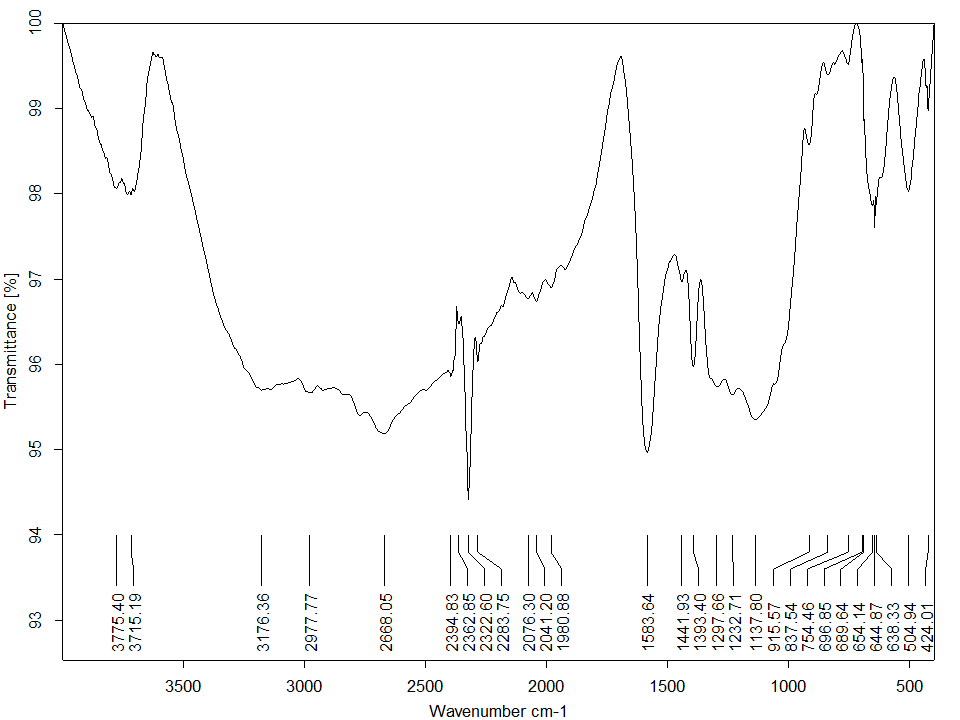 |
| --- |
| 1. OPAC prepared at 700 °C |
| 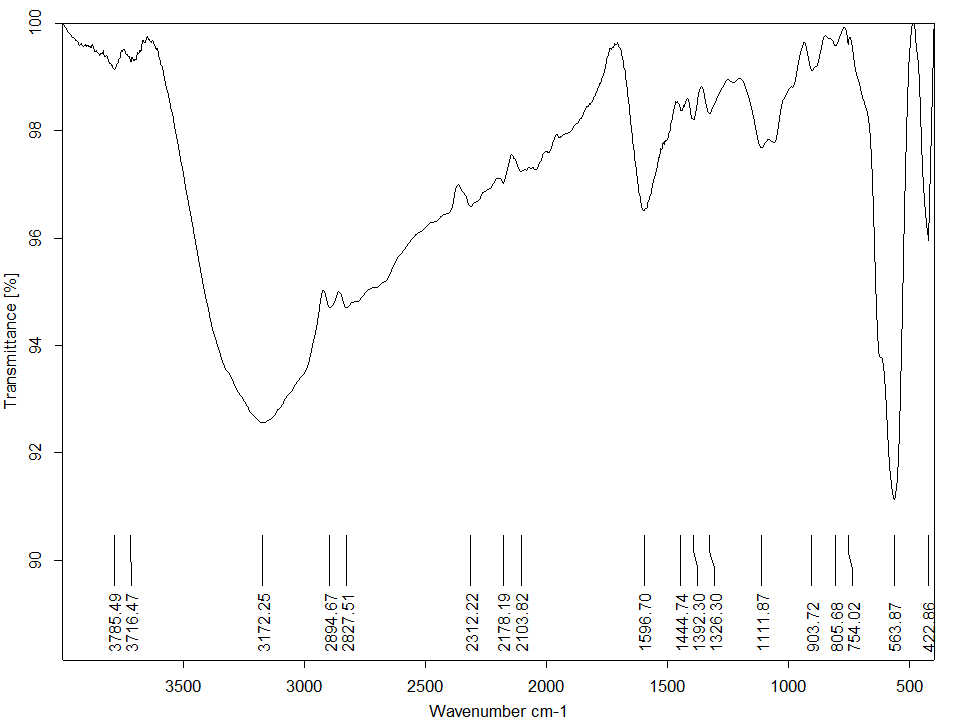 |
| 1. MG-OPAC prepared at 700 °C |
| 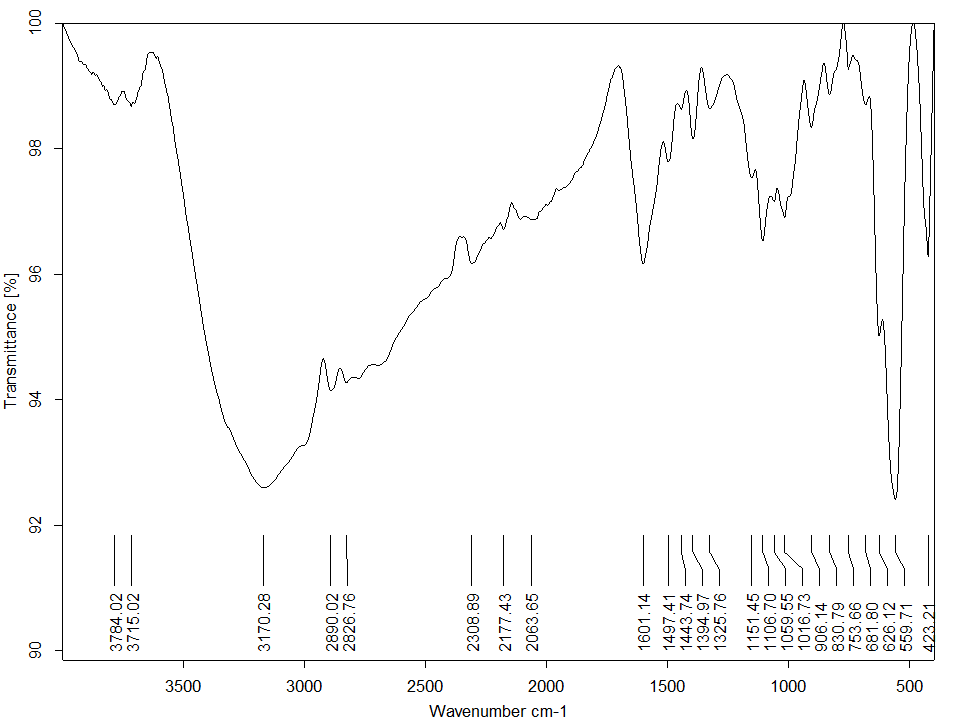 |
| (c) AO7 dye adsorbed on MG-OPAC |

**Figure S2**. The FTIR of (a) OPAC prepared at 700 °C, (b) MG-OPAC prepared at 700 °C, (c) AO7 dye adsorbed on MG-OPAC prepared at 700 °C.

| 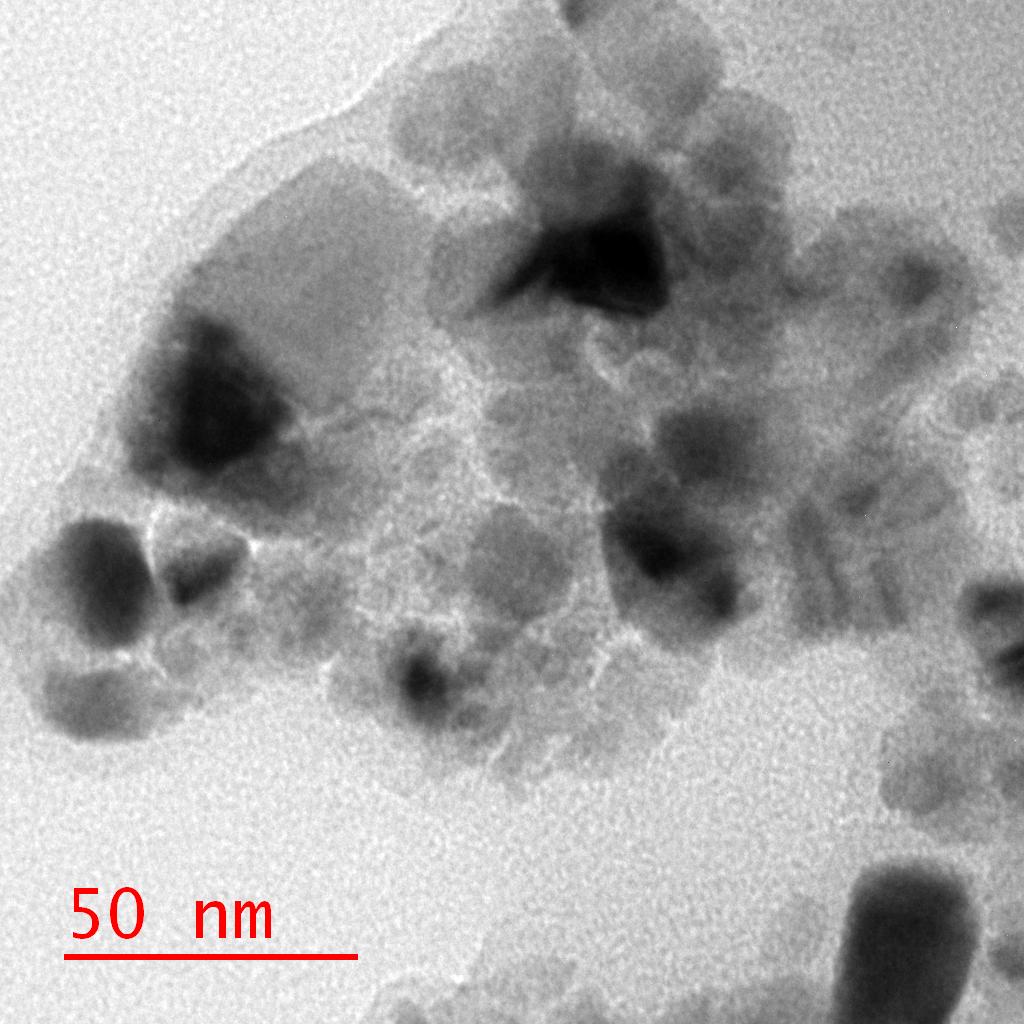 |
| --- |
| (a) |
| 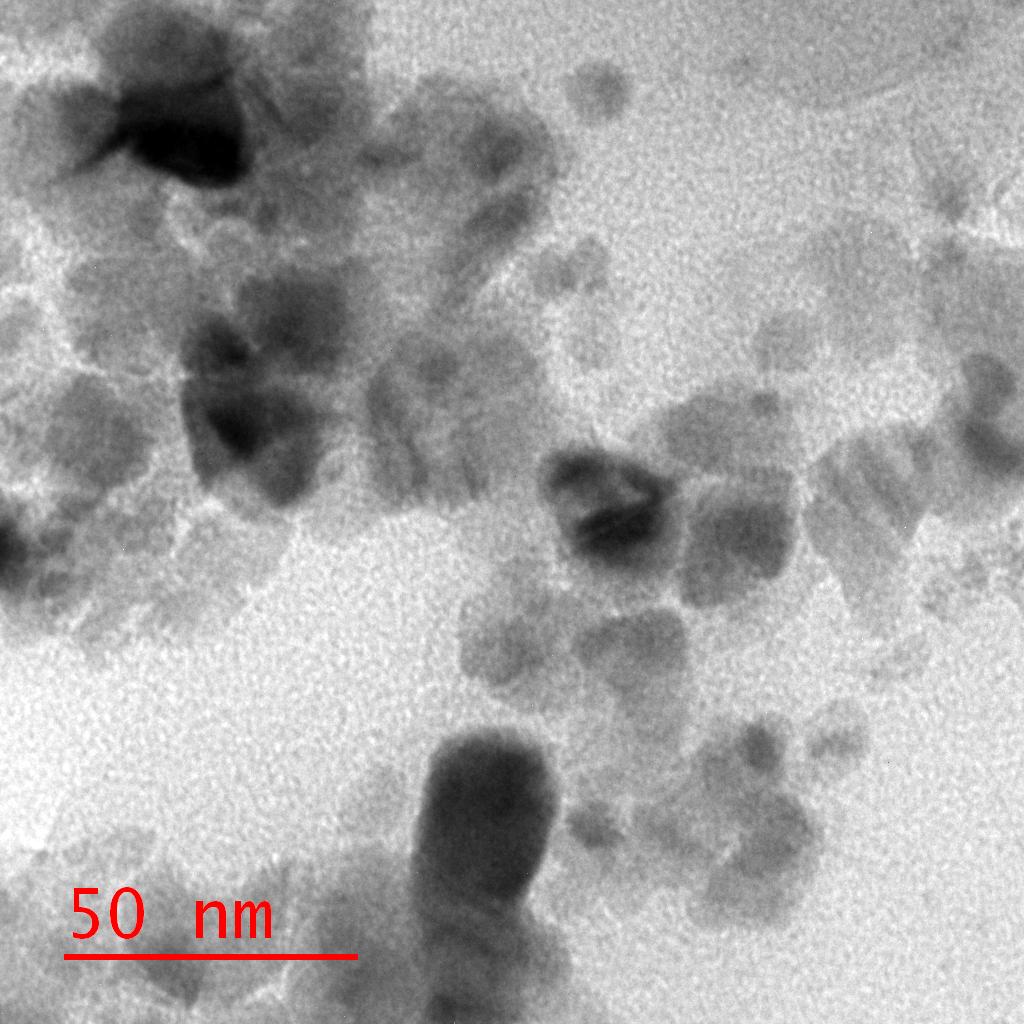 |
| (b) |

**Figure S3**. TEM analysis image of MG-OPAC prepared at 700 °C.
